# Investigation of boron-doped graphene oxide anchored with copper sulphide flowers as visible light active photocatalyst for methylene blue degradation

**DOI:** 10.1038/s41598-023-36486-6

**Published:** 2023-06-12

**Authors:** Ahmad Farhan, Muhammad Zahid, Noor Tahir, Asim Mansha, Muhammad Yaseen, Ghulam Mustafa, Mohammed A. Alamir, Ibrahim M. Alarifi, Imran shahid

**Affiliations:** 1 Department of Chemistry, University of Agriculture, Faisalabad, 38040 Pakistan; 2 Department of Chemistry, G. C. University, Faisalabad, 38040 Pakistan; 3 Department of Physics, University of Agriculture, Faisalabad, Pakistan; 4 Department of Chemistry, University of Okara, Okara, Pakistan; 5 Department of Mechanical Engineering, College of Engineering, Jazan University, Jazan, 45142 Saudi Arabia; 6 Department of Mechanical and Industrial Engineering, College of Engineering, Majmaah University, Al-Majmaah, Riyadh, 11952 Saudi Arabia; 7 Environmental Science Centre (ESC), Qatar University, P.O. Box 2713, Doha, Qatar

**Keywords:** Photocatalysis, Mechanical and structural properties and devices, Synthesis of graphene

## Abstract

The non-biodegradable nature of waste emitted from the agriculture and industrial sector contaminates freshwater reserves. Fabrication of highly effective and low-cost heterogeneous photocatalysts is crucial for sustainable wastewater treatment. The present research study aims to construct a novel photocatalyst using a facile ultrasonication-assisted hydrothermal method. Metal sulphides and doped carbon support materials work well to fabricate hybrid sunlight active systems that efficiently harness green energy and are eco-friendly. Boron-doped graphene oxide-supported copper sulphide nanocomposite was synthesized hydrothermally and was assessed for sunlight-assisted photocatalytic degradation of methylene blue dye. BGO/CuS was characterized through various techniques such as SEM–EDS, XRD, XPS, FTIR, BET, PL, and UV–Vis DRS spectroscopy. The bandgap of BGO-CuS was found to be 2.51 eV as evaluated through the tauc plot method. The enhanced dye degradation was obtained at optimum conditions of pH = 8, catalyst concentration (20 mg/100 mL for BGO-CuS), oxidant dose (10 mM for BGO-CuS), and optimum time of irradiation was 60 min. The novel boron-doped nanocomposite effectively degraded methylene blue up to 95% under sunlight. Holes and hydroxyl radicals were the key reactive species. Response surface methodology was used to analyze the interaction among several interacting parameters to remove dye methylene blue effectively.

## Introduction

Water has always been one of the most important and diverse features of all living forms, and contamination of water resources is a problem that demands careful consideration^1^. Industrial effluent, including various factors like pesticides, herbicides, dyes, and organic pollutants, is a significant source of water pollution^2^. A trivial amount of these pollutants may significantly impact the ecosystem and can impact climate change. Different chemical substances are utilized in dyeing processes and various industrial units, releasing effluents, including colourful, non-biodegradable, and partly hazardous chemicals and dyes^3,4^. This wastewater is a major contributor to methane emissions (about 10%), leading to a rise in temperature and causing global warming. Because of this, wastewater must also be cleaned before being released into rivers to have the least environmental damage^5,6^. Among the leading causes of water pollution are non-biodegradable chemicals and soluble dyes^7^.

Overpopulation, rising food demand, and increased industrialization are the main reasons for wastewater pollution^8,9^. The threat of water contamination is growing day by day. Water contamination is a concern in underdeveloped nations, with most of the country’s water in lakes and rivers contaminated^10,11^. Many rural communities worldwide face the twin challenges of water scarcity and water pollution by microbiological and chemical pollutants^12,13^. Industrial effluents are primarily generated in the textile industry, where massive volumes of water are used at each step of numerous operations, especially during dyeing.

According to United Nations Organization(UNO), half of the population of developing countries suffers from health problems caused by microbiologically or chemically contaminated drinking water^14^. The main concern is the microbiological cleanliness of consumable water in general. Waterborne diseases kill 5 million people annually. Human consumption of freshwater has been reduced to 0.01%, with the earth’s surface having only 3% freshwater reserves. Population growth increased freshwater needs. As population growth accelerates, there will be a severe freshwater shortage for a sustainable life^15^.

Advanced oxidation processes (AOPs) are used in water treatment facilities to eliminate pathogens and disease-causing micro-organic contaminants. (AOPs) are considered cutting-edge techniques. The first suggestion to employ AOPs for drinking water filtration was made in 1980. Later, scientists began viewing them as a potential oxidizing treatment for different types of wastewater^16,17^. But, every method comes with its merits and demerits. The heterogeneous semiconductor photocatalysis is an environmentally friendly green technique utilizing sunlight as a potential source for the activation of semiconductor materials for pollutants degradation^18^. The pollutants in the water that are the subject of the treatment are mineralized and, in some circumstances, chemically removed as a consequence of the interactions between all of the radicals and the production of further reactive species such as superoxide and H_2_O_2_. When oxidizing agents (such, as hydroxyl radicals are generated locally. AOPs are used as tertiary methods, these generate unstable hydroxyl radicals that degrade quickly. Heterogeneous photocatalysis employs semiconductor metal oxides and sulphides. These materials produce electron–hole pairs as soon as they are exposed to ultraviolet light or sunlight irradiation. But, these charge carriers rapidly recombine and limit their efficiency^19^. To enhance the photo-efficiency of these metal sulphides, they are coupled or immobilized with carbon-based support materials. This development of a new interface helps in the development of new sunlight-active support-based systems for wastewater treatment^20,21^.

Due to high setup costs and low throughput, this wastewater treatment option has not been studied nearly as extensively as others^22^. Metal sulfides are significant photocatalysts that may stimulate several proper redox processes under moderate circumstances^23^. Metal sulfides have lower bandgaps than metal oxides, allowing them to use more visible light^24^. Many transition-metal sulfides, like zinc sulfide (ZnS), cobalt sulphide (CoS), iron sulphide (FeS), and cadmium sulphide (CdS), have been extensively studied as photocatalysts^25–27^. This feature makes sulphides the most common visible light-active semiconductor photocatalysts^24^. In recent times CuS has gained much importance due to its capability to eliminate contaminants from polluted water. Copper sulfide has demonstrated significant reactivity and high reusability in the decomposition of various dyes in the presence of solar light with enhanced sunlight^28,29^.

Among metal sulfide photocatalysts, copper sulfide (CuS) has shown significant activity in environmental remediation^30^. CuS has been widely researched in oxidizing dye waste water owing to its unusual chemical, physical, electronic, and optical features^31^. CuS is a significant p-type semiconducting with magnificent photocatalytic properties and can be prepared with various morphologies, including nanospheres, nanoplates, nanorods, and nanocubes based on aggregation and synthesis methods. The unique morphology of CuS constructed hierarchically via easy chemical solution deposition at room temperature revealed hollow nanocatalysts. While some prepared hydrothermally were nanoplates with higher quantum efficiency and larger specific surface area^32^. The performance of CuS has been improved over the years with various strategies like doping and coupling with other semiconductor materials. These CuS-based composites showed excellent photodegradation efficiency, as previously reported by various researchers on CuS-based composites^33^.

Graphene oxide (GO) is an excellent support material for the fabrication of visible light-active heterogeneous nanocomposites, as an introduction of GO support reduces the recombination time of light-induced charge carriers^34^. GO acts as a sink for electrons coming from the conduction band of CuS, hence suppressing their recombination by providing them with a longer path. The defects created due to B-doping in the π-framework of GO enhanced its surface area and provided more adsorptive sites for pollutant molecules. Various atoms have been doped in graphene oxide, like nitrogen, phosphorus, iodine, and boron^35–37^. Among them, boron doping is most important because due to similar in size as it occupies the lattice framework. The doping of boron increases the chances of charge resistance^38^.

Boron-doped graphene oxide (BGO) has widely been investigated for environmental applications and energy devices because the B-doping creates more active sites on GO edges^39^. Limited research has been reported on the photocatalytic degradation of pollutants using B-doped graphene oxide coupled semiconductor materials, as doping GO with non-metal atoms is a very selective process^40,41^. Hence, more insight into BGO-based nanocomposites is required to unlock their efficiency in degrading different pollutants. The present research study reveals an effective two-step procedure including non-metal (B) doping on carbon-based support GO in the first step and subsequently coupling with CuS hydrothermally, synthesizing BGO-CuS. This nanocatalyst is prepared for the first time to the best of our knowledge was assessed for its photocatalytic efficiency towards the degradation of dye methylene blue (MB) under sunlight irradiation. The doping of Boron in graphene oxide has significantly reduced bandgap and improved electrical conductivity. The primary emphasis remained on the development of a photocatalyst that showed improved photocatalytic activity under sunlight. Numerous techniques, such as FT-IR, SEM–EDS, XPS, BET, XRD, UV–Visible DRS, and PL were utilized for the detailed characterization of the composite. Various parameters (pH, catalyst dosage, oxidant dose, time, and initial dye concertation) were optimized to check the photocatalytic efficiency of the prepared composite. The synergistic effect between the CuS and BGO helped enhance the photocatalytic efficiency under sunlight.

## Experimental and synthesis

### Materials and reagents

Sodium Nitrate (NaNO_3_, > 99%), Potassium Permanganate (KMnO_4_, > 99%), Copper(II) Nitrate Trihydrate (CuH_2_N_2_O_7_, > 99%) Sulfuric Acid (H_2_SO_4_, 98%), Hydrogen Peroxide (H_2_O_2_, 35w/w%), ethanol (CH_3_CH_2_OH, 95.6%), Boric acid (H_3_BO_3,_ 99.5%), and Thiourea (CSN_2_H_4_, 96%) were purchased from Sigma Aldrich (USA). The dye methylene blue was obtained from the Fischer Scientific company. The graphite powder was obtained from Scharlau (Spain). Boric acid, Copper (II) Nitrate trihydrate, and Thiourea were obtained from Daejung (South Korea). Sodium Nitrate and potassium permanganate were obtained from Merck. All the compounds were of analytical quality, and none underwent further purification before usage. Throughout the research project, distilled water was used for carrying out all reactions.

### Synthesis of GO, BGO, and GO-CuS

A modified version of Hummer’s technique was employed to produce graphene oxide (Fig. S1), as previously reported by our research group^42^. Prepared GO was dispersed ultrasonically and mixed in an aqueous solution containing boric acid in a 1:3 ratio, resulting in boron-doped graphene oxide synthesis^43^. The hydrothermal method was used to make the CuS/GO nanocomposites, as previously reported by Saranya et al.^44^. All the synthesis details are presented in the synthesis section of (supplementary information).

### Synthesis of BGO-CuS

**Figure 1:**
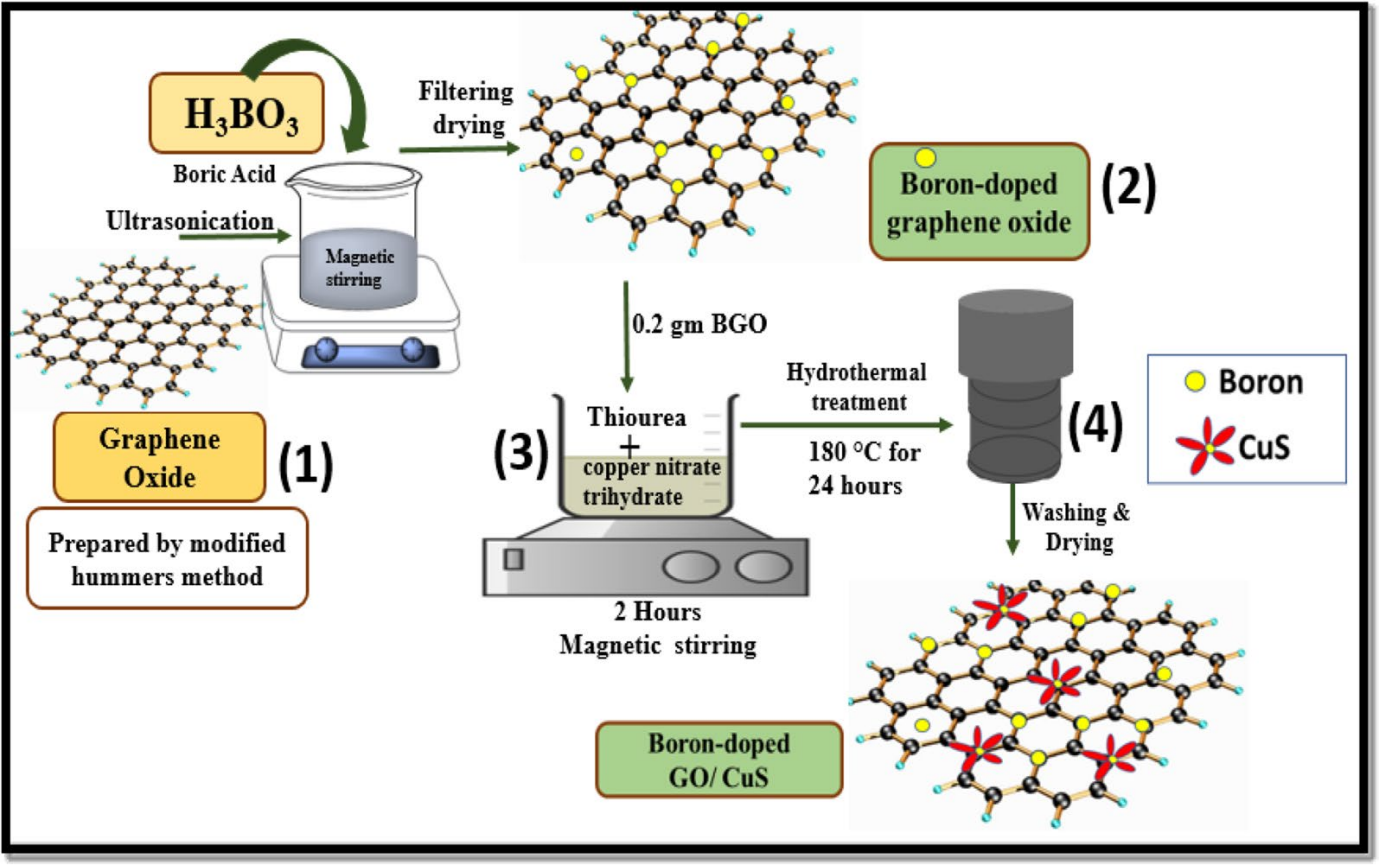
Schematic scheme representing a hydrothermal synthesis of BGO-CuS.

The binary boron-doped graphene oxide/copper sulphide (BGO-CuS) nanocomposite was prepared by the ultrasonication-assisted facile hydrothermal method. The hydrothermal technique was used to make the CuS/BGO nanocomposites. Firstly, a uniform suspension of boron-doped graphene oxide was obtained by dissolving 0.02 g in 50 mL of distilled water. The mixture was then ultrasonicated for 30 min to obtain a uniform suspension. The appropriate amount of copper nitrate (Cu(NO_3_)_2_ was added to 40 mL distilled water, and the solution was constantly stirred until a blue solution formed. After that, thiourea was added under constant magnetic stirring. After the solution had been well mixed, 0.2 g of already properly dispersed BGO was added to the mixture in a dropwise manner in about an hour. After vigorous magnetic stirring for another two hours, the solution was poured into an autoclaved, which was kept for 24 h at 180 °C in an oven. BGO/CuS nanocomposite was obtained by centrifuging the black precipitate with distilled water and ethanol after drying it in an oven. Figure 1 depicts a schematic diagram of a novel composite synthesis.

### Characterization and equipment

A scanning electron microscope (SEM) equipped with energy dispersive spectroscopy (EDS) was used to characterize the morphology, elemental analysis, and microstructure of as-produced GO-CuS and BGO-CuS (SEM–EDX; FEI NOVA 450 NANOSEM). The crystalline characteristics of each powder sample were evaluated using XRD (controlled by Bruker D8 Advanced equipment) throughout a wide range of 2θ values, which extended from 5° to 80°. Using FTIR, the existence of several functional groups on the surfaces of manufactured photocatalysts can be observed (FTIR, Thermo Nicolet). The bandgap measurement of the prepared composites was carried out using a UV–VIS spectrophotometer (Cecil CE 7200) in the 200–800 nm range. THE XPS analysis for the elemental state analysis was done using the (Escalab 250-XPS system, Thermo Fischer Scientific UK). A Brunauer–Emmett–Teller (BET) analysis (Nova 2200e-Quantachrome) was used to measure the surface area and surface porosity of prepared nanocomposites. The photoluminescence spectra were studied using (Shimadzu RF-5301PC), with an excitation wavelength of 325 nm.

### Analysis of photocatalytic degradation

For one hour in batch mode, BGO-CuS, GO-CuS, and CuS nanocomposites were used to study the photocatalytic degradation of the cationic dye MB. These tests have proceeded in visible light. Irradiation to examine the nanocomposites in a range of concentrations to optimize conditions of several working parameters for effective pollutant (MB) removal. The influence of several factors on MB photodegradation was investigated, including pH (ranging from 2 to 9), catalyst dose (ranging from 10 to 100 mg/100 mL), oxidant dose (ranging from 5 to 40 mM/100 mL) initial dye concentration (ranging from 5 to 50 ppm), and irradiation period (ranging from 10 to 60 min). A blank or control experiment was placed with each optimized parameter under sunlight. The blank with no catalyst and oxidant added to the MB solution showed no noticeable degradation, indicating that photolysis was insignificant for the MB solution. To facilitate the photocatalytic degradation of methylene blue in the presence of sunlight, the required amounts of catalysts were sonicated in 100 mL of dye solution. The whole setup was placed directly under sunlight from 12 to 2 pm in natural daylight with constant shaking of dye-containing solutions in an orbital shaker. The solar power meter, model number SM206, was used to measure the intensity of the sunlight, while a light meter was used to measure the brightness (HS1010A). To achieve a state of adsorption–desorption equilibrium in the solutions before they were exposed to sunlight, the solutions were manually agitated for about thirty minutes in the dark. After determining the photocatalysts' capability for adsorption, the dye solutions were subjected to sunlight irradiation for sixty minutes. After each run, 5 mL of treated dye solution was centrifuged to separate the nanocomposites. The absorbance of the treated dye solution was measured using a UV–VIS spectrophotometer at 665 nm wavelength to determine the dye's concentration after treatment under sunlight irradiation. To test the rate of dye deterioration in both the presence and absence of light, an empty beaker containing dye solution was stored in the dark and direct sunshine. Using the following Eq. (1), we determined the percentage of dye degradation.

1 $$\% {\text{Degradation}} = 1 - \frac{{\text{A}}}{{{\text{Ao}}}} \times 100$$

The initial absorbance, denoted by (Ao), is compared to the absorbance following treatment by solar light, represented by A. The pH level was kept stable with a pH meter and a variety of HCl and NaOH solutions of varying molar concentrations (Ohaus ST3100, USA). Catalysts were reused five times, each time under optimum circumstances under sunlight with a freshly prepared 10 ppm MB solution, to investigate their reusability. This aimed to determine the maximum utilization of BGO-CuS to determine its cost-effectiveness.

## Characterization

### FTIR Analysis

**Figure 2:**
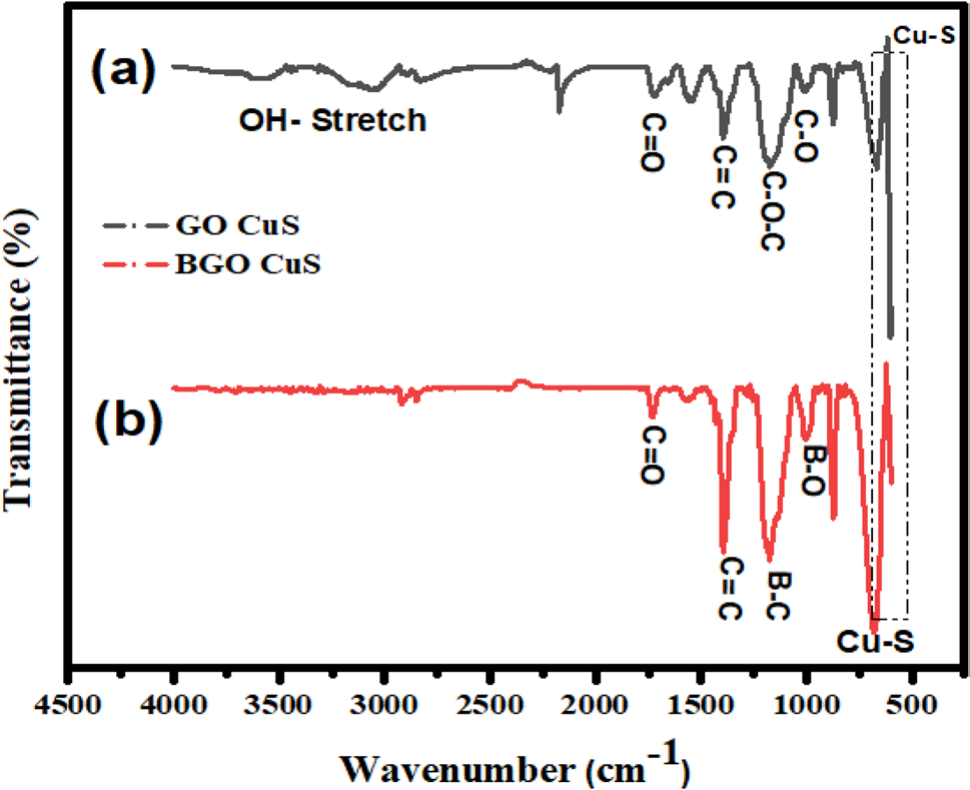
FTIR Spectra (**a**) GO-CuS and (**b**) BGO-CuS.

The Fourier transform infrared spectroscopy approach identifies functional groups on the catalyst surface. Figure 2 shows the FTIR data obtained for the B-doped and undoped samples. Adsorption of CuS in the stretching mode took place at 628 cm^−1^. GO exhibited vibrational patterns revealing the presence of several groups like C=C (1421 cm^−1^), C=O (1632 cm^−1^), O–H (3231 cm^−1^), and C–O–C (1382 cm^−1^). The peak O–H (3231 cm^−1^) shows the presence of the hydroxyl group in the GO. When a non-metal like Boron is doped into GO, the strength of the -OH peak is somewhat reduced, as depicted in Fig. 2. The O–H stretching vibrations of adsorbed water molecules and structural OH groups cause a broad and wide band at 3403 cm^−1^ in the FT-IR spectra of as-synthesized GO.

The peak that can be found around 1620 cm^−1^ is linked to the bending vibrations mode of C=O groups. The vibration mode absorbs sound at a frequency of 1399 cm^−1^, whereas the frequency of the C–O stretching is 1077 cm^−1^^45^. The asymmetric B–O stretch of B–O–B connections between major units may be attributed to the band at 1038 cm^−1^ in the spectra of B-doped GO^46^. These bonds connect one trigonal and one tetrahedral Boron atom. The band at roughly 1185 cm^−1^, which is placed there, represents the Boron–Carbon stretching vibration. The B–C band is most often found in the frequency range of 1050–1200 cm^−1^. It was hypothesized that the number of B atoms in the sample would affect the frequency of Boron–Carbon stretching. The B replacement in the carbon network efficiently downshifts the variances because B–C has a substantially lower force constant than the C–C bond. This is because the force constant of BAC is much smaller than that of C–C^47^. On the other hand, the increase in the frequency of B–C stretching is most likely due to the higher carbon content. The BGO-CuS transmittance peaks are lesser in intensity as compared to GO-CuS, endorsing its effective insertion of dopant in the support matrix.

### XRD analysis

**Figure 3:**
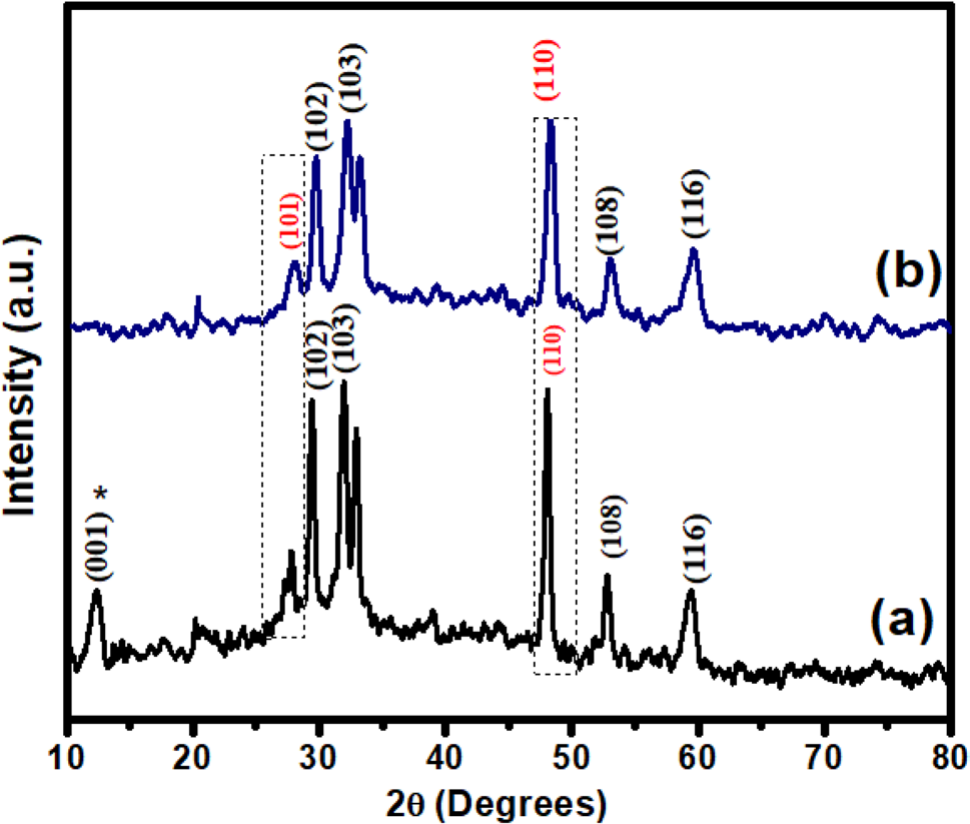
XRD spectra of (**a**) GO-CuS and (**b**) BGO-CuS.

As-prepared CuS exhibited diffraction peaks at 2θ values of 25.6°, 29.8°, 33.6°, 48.5°, 52.5°, 60.2° indexed at crystallographic planes (101), (102), (103), (110), (108) and (116) correspondingly (JCPDS card No. 00-001-1281), confirming the hexagonal phase structure of CuS nanoparticles^48^. The addition of graphene oxide did not alter the phase structure of the CuS hybrids since all of them showed identical peaks to pure CuS. But GO showed a broadening of the peak at the 2θ value of 20.1° in the GO-CuS composite, while the characteristic peak of GO at 11.71° (001) degrees is present in the GO-CuS composite^45^. Doping of boron is indicated by a peak shift at 2θ values of 26.4° and 49.2° at relative indices of (101) and (110) CuS planes, respectively, towards higher angles. This suggests that CuS has combined and coupled effectively with BGO. The shift of peaks towards higher angles is due to distortion in the lattice interface, as shown in Fig. 3^40^. Moreover, the GO-CuS composite shows a broad and less intense peak at 2θ = 11.71°, but the disappearance of GO peak (001) in BGO-CuS is linked with the removal of oxygen-containing functional groups from BGO-CuS during hydrothermal treatment^37,49^. Debye–Scherrer formulae determined the average particle size in Eq. (2):

2 $$D = 0.9\lambda /\beta \cos \theta$$

D is the crystallite size, is the wavelength of CuK (1.54 A), and describes the intensity at the full-width half maximum of the diffraction line, which is represented in radians and is Bragg’s angle. According to the results, BGO-CuS, GO-CuS, and CuS have an average size of 26.2 nm, 53.21 nm, and 93.45 nm, respectively. Since the incorporation of boron reduces nucleation and grain formation, the lower crystallite size may be understood as the result of the improved growth of copper sulfide onto support media despite their constrained dimensions, which may deform the host lattice^50^.

### SEM–EDS analysis

**Figure 4:**
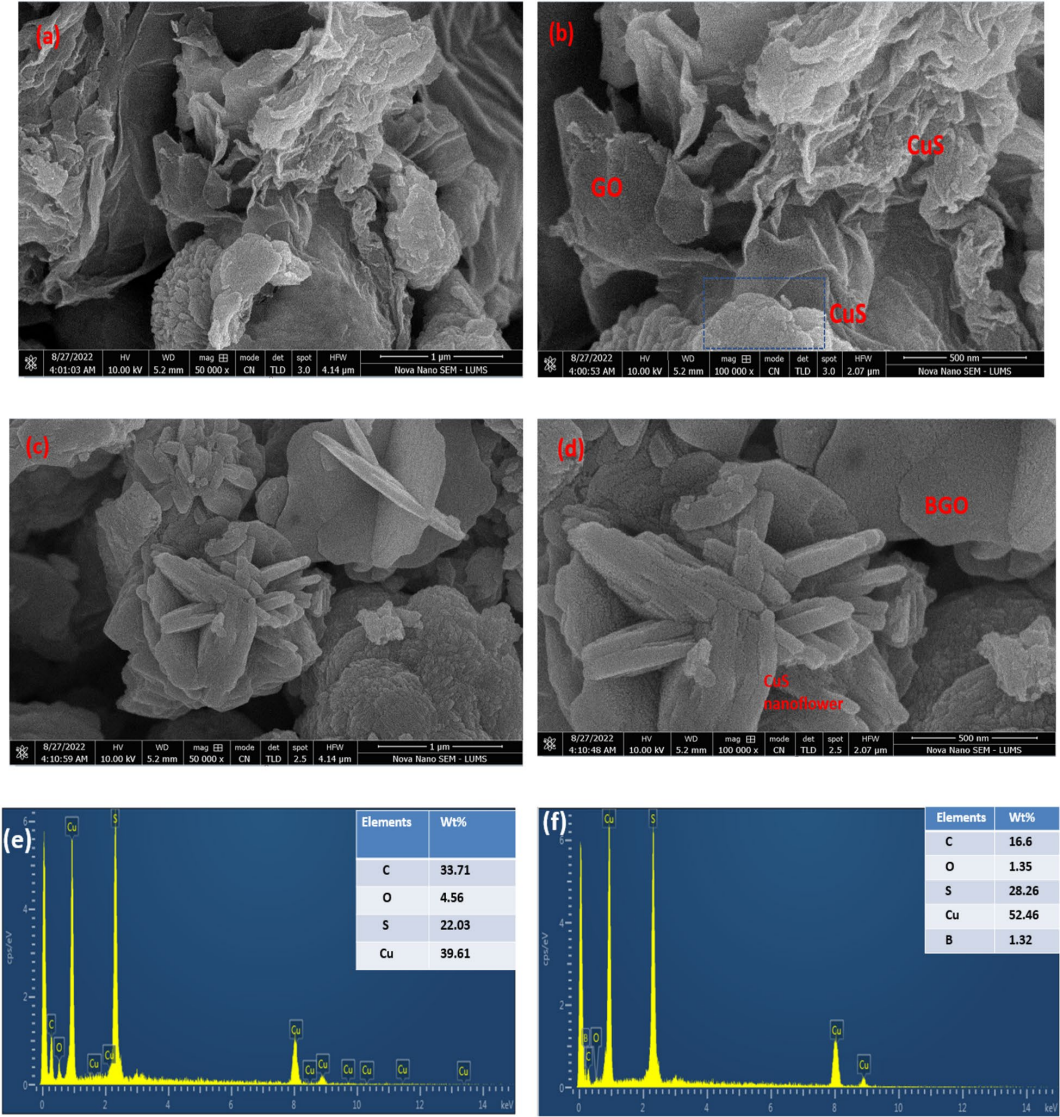
SEM images of (**a** and **b**) GO-CuS flowers and (**c** and **d**) BGO-CuS (**e** and **f**) EDX of GO-CuS and BGO-CUS.

On the surface of GO nanosheets, microsphere formation occurred in situ, with fewer microspheres present at higher initial GO contents. The ripening process during the hydrothermal process initiated nucleation. Secondly, these elementary particles cluster together, and CuS crystals form as their nuclei develop preferentially in the same direction. Primary atomic forces, such as van der Waals interactions, lead to nanoparticle aggregation^44^. The nano-platelets aggregated with one another, leading to the development of self-assembled CuS composites with bigger, more varied flower-like topologies, revealed in Fig. 4a, b. High temperatures aid in the creation of uniform CuS nanostructures. Well-defined morphologies may be produced by controlling the growth and nucleation phases^45^. Doping of Boron in graphene oxide resulted in more uniform sheet-like structures, as seen in Fig. 4c and d. These uniform B-GO sheets have enhanced surface area for anchoring CuS flowers, helping them prevent the recombination of e^−^/h^+^ pairs. The porous and visible granular appearance of BGO confirms its synthesis and incorporation of boron atoms between GO sheets. The sheet structure arising in a hierarchical arrangement prevents CuS from restacking and aggregation. This type of morphology provided better support for the formation of CuS flowers from BGO cavities providing them with enhanced surface area and better adsorption sites^48^. This ultimately resulted in better transport of electrons and holes among the interfaces. The addition of boron atoms in the GO honeycomb lattice has resulted in strong linkages in graphene oxide sheets with more interconnected channels^51^.

The elemental composition revealing purity, elemental position, and uniform distribution of several essential elements in BGO-CuS was estimated by energy-dispersive X-ray spectroscopy (EDS). It confirmed the presence of boron, carbon, oxygen, copper, and sulfur, with the inset showing weight percentage in BGO-CuS. The absence of other impurities in samples is also evident from EDS^52^. The elements by weight percentage are carbon (16.6), Oxygen (1.35), Sulfur (28.26), Copper (52.46), and Boron (1.32), as shown in Fig. 4e and f. The EDS spectra of BGO-CuS show the presence of boron as compared to undoped GO/CuS. The individual elemental mapping of BGO-CuS has been presented in Fig. S2 of supplementary information.

### XPS analysis

**Figure 5:**
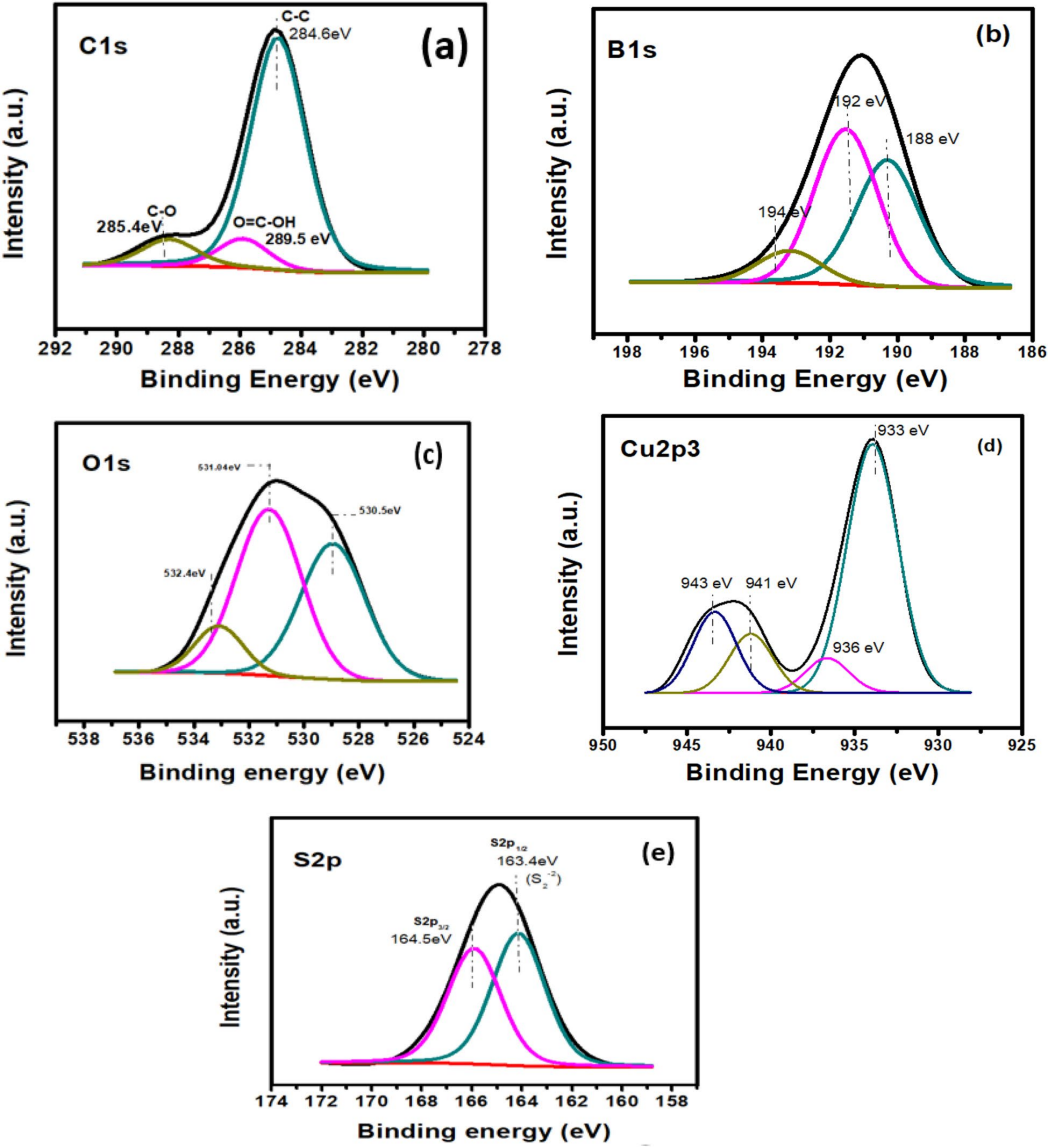
High-resolution XPS spectra of (**a**) C1s (**b**) B1s (**c**) O1s (**d**) Cu2p3 (**e**) S2p in BGO-CuS nanocomposite.

The elemental and chemical composition of prepared BGO-CuS is examined through X-ray photoelectron spectroscopy (XPS). The high-resolution spectra of major elements are presented in Fig. 5. A C1s peak at 285.4 eV is related to adventitious carbon. The peaks arising at 283.1 eV and 284 eV binding energies in C1s high-resolution spectra are those of Sp2 and Sp3 hybridization of a single C peak^53^. The deconvoluted high-resolution spectrum of the Cu2p peak shows two peaks at 933.5 eV and 936 eV binding energies, linked to 2p_3/2_ (Cu) and 2p_1/2_ states of Cu(II) ions, respectively. A small satellite peak that appears at 941 eV is characteristic of copper ions. The B1s show two strong peaks. The main peak (194.2 eV) is related to boron that is interstitially interlaced in graphene structure, and another peak at 192.6 eV is due to B–O–B groups that are typical of boron atoms in H_3_BO_3_^49^. A small peak at 188 eV relates to B-atoms occupying O-sites in graphene oxide lattice. The O1s spectra show two peaks at 530 eV and 533 eV owing to the hydroxyl group and water molecules adsorbed over the catalyst surface, respectively. The deconvoluted peaks arising at 163.0 eV and 167.1 eV binding energy is due to S2p_3/2_ and S2p_1/2_ of S^2-^ required for forming CuS^53^.

### BET analysis

**Figure 6:**
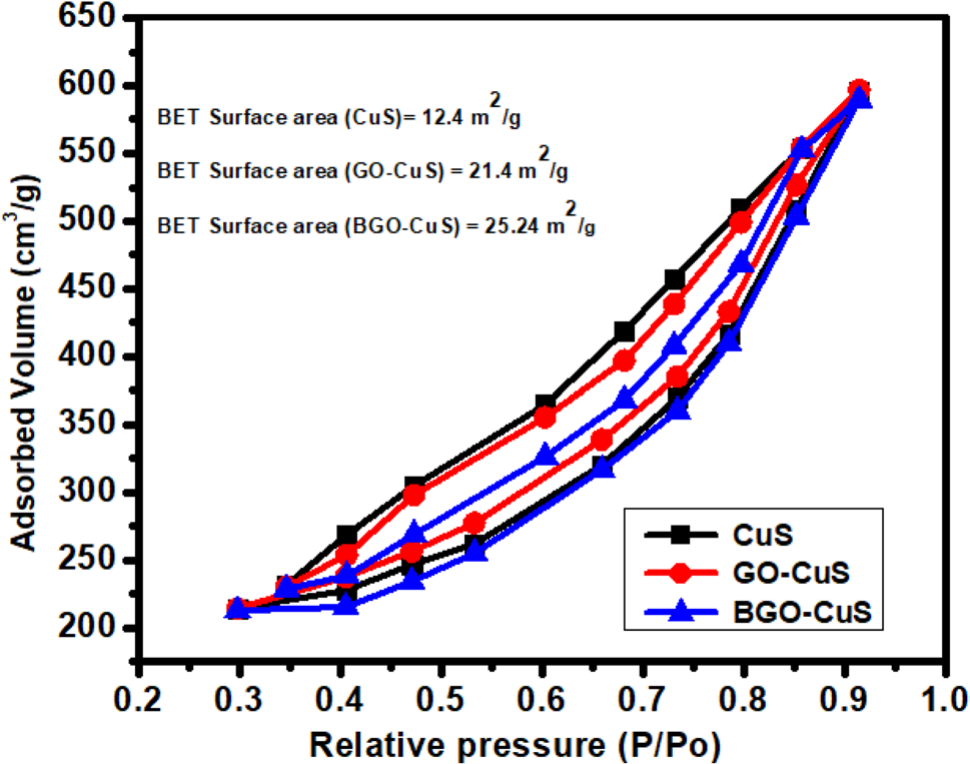
BET surface area analysis of BGO-CuS, GO-CuS, and CuS.

The surface area of a nanocomposite is the most critical factor in determining its photocatalytic activity. The performance of a photocatalyst in the photodegradation process is highly dependent on its surface area. B-GO/CuS, GO/CuS, and CuS BET adsorption/desorption isotherm is shown in Fig. 6. A type IV isotherm with a distinctive hysteresis loop was observed for all nanocomposites according to IUPAC classification. This loop suggests that prepared nanocomposites have lamellar sheet-like pore structures and mesoporous nature. Using the nitrogen gas adsorption data, the specific surface area was calculated using the Brunauer–Emmett–Teller technique, yielding values of 25.24 m^2^/g for BGO/CuS and 21.4 m^2^/g for B-GO/CuS. A larger surface area indicates that the BGO/CuS nanocomposite was successfully synthesized. Doping has successfully increased the surface area of the catalyst, providing more active sites and enhancing degradation efficiency. More molecules of pollutants are adsorbed on the surface of the catalyst, indicating that more active radicals are generated as a result. This immediately improves photocatalyst efficiency by making more light absorption available^54^.

### Optical analysis

Using UV–visible spectra, it was possible to calculate the bandgap of CuS, GO-CuS, and BO-CuS. Estimates of bandgap energies of the catalysts were obtained by the proposed formula presented in Eq. (3)

3 $$\left( {\alpha hv} \right)^{{2}} = \left( {{\text{hv}} - {\text{Eg}}} \right)$$

**Figure 7:**
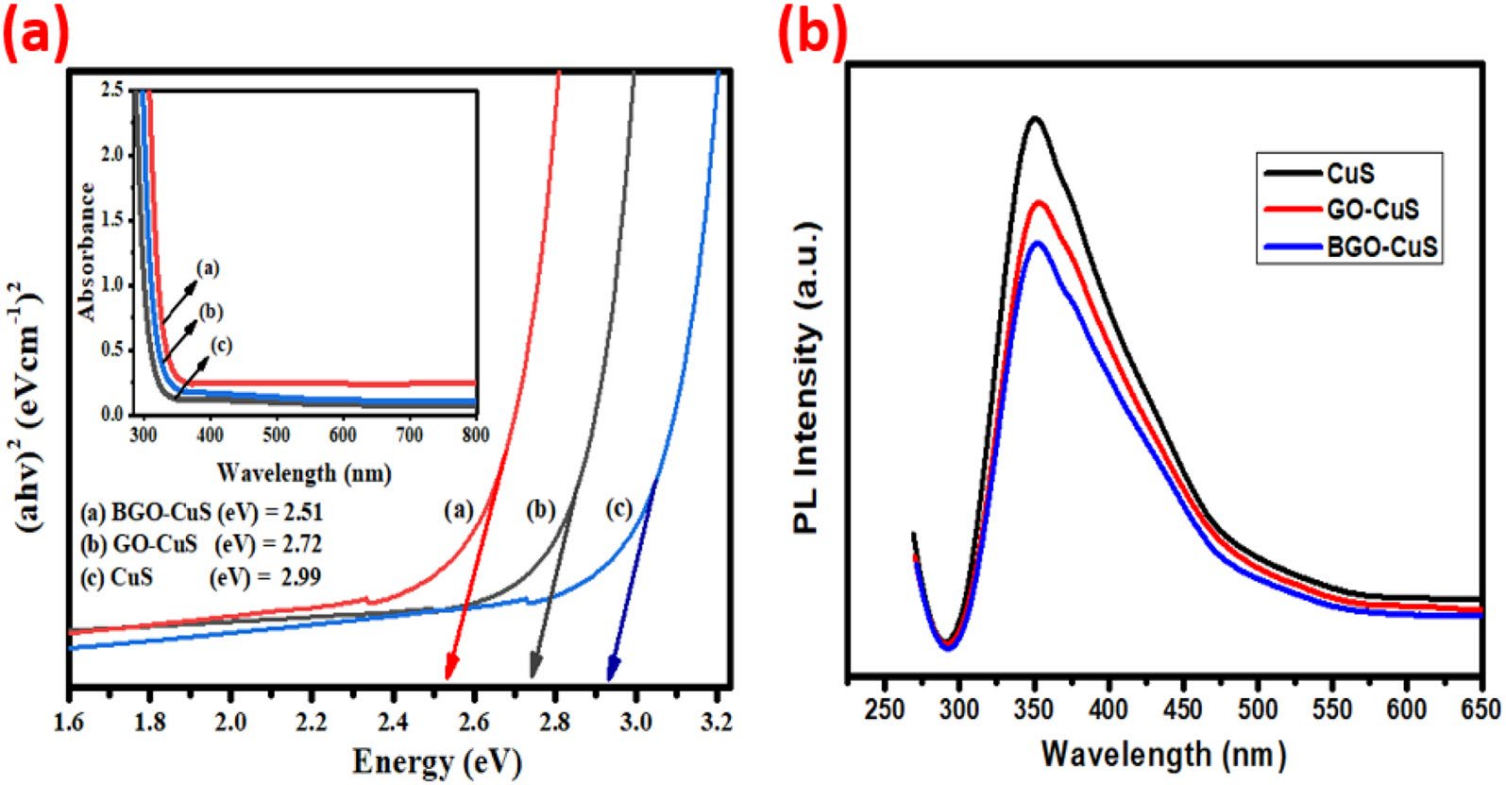
﻿(**a**) Bandgap energies estimation by Tauc plot method with inset showing the UV–VIS absorption spectra of all nanocomposites (**b**) PL spectra of nanocomposite.

The bandgap of the copper sulfide is around 2.90 eV. GO-CuS, on the other hand, GO/CuS had a bandgap lowered from 2.90 eV to 2.7 eV due to composite formation with graphene oxide. Because of the narrowing of the bandgap, the photocatalytic activity of GO-CuS became more effective. As a result of the high bandgap, a more significant amount of energy is required for the excitation of e− as it travels from the valence Band (VB) to the conduction Band (CB)^55^. The large energy band is the primary factor that involves the limited amount of photocatalytic activity of copper sulfide. While comparing GO-CuS and BGO-CuS bandgaps, the figure depicted that doping of Boron in GO resulted in a reduction of band gap (2.5 eV), which resulted in enhanced photocatalytic activities of BGO-CuS Fig. 7a. The doped graphene oxide- metal sulphide-based nanocomposites can be improved easily by tuning the edge defects and surface modifications of doped graphene sheets. This is owing to the addition of oxygen functionalities in basal planes changing the Sp2 configuration to tetrahedral Sp3 configuration. These doped GO sheets act as a transport barrier for charge carriers. This ultimately changes the electronic properties of coupled materials by changing their energy levels and the appearance of new energy states from the functionalized oxygen groups^45^.

Additionally, the doped graphene oxide-supported semiconductor nanocomposites are supposed to exhibit excellent photodegradation efficiency in the visible region of the solar spectrum. The presence of GO narrows down the bandgap and enhances the visible light response^56^. Graphene oxide serves the purpose of an electron acceptor reservoir by capturing and retaining electrons from the metal oxide’s conduction band. This ultimately leads to the suppression of the recombination rate of charge carriers by separating charge carriers for longer periods, enhancing the photostability and photoefficiency of catalysts^40^.

### Photoluminescence analysis

PL spectra were used to analyze the behaviour of electron–hole pairs created by light irradiation, including their isolation and recombination. Recombination between e^−^/h^+^ pairs is slower for lower PL intensities and faster for higher PL intensities^57^. PL emission spectra of B-GO/CuS, GO/CuS, and CuS were recorded to examine the electron–hole recombination behaviour of B-doped and undoped nanocomposites, as shown in Fig. 7b. The rapid decrease in PL intensity seen in boron-doped nanocomposite indicates the emergence of additional active sites due to the interstitial effects of boron doping. In general, the intensity of the PL emission decreases from BGO/CuS to GO/CuS to CuS. As compared to other nanocomposites, B-GO/CuS has superior catalytic activity due to its lower PL intensity, which occurs when the electron–hole pairs are separated for a longer time. The more active sites provide more surface area to bind with pollutant materials^58^.

## Effect of operational parameters

### pH effect on MB degradation

**Figure 8:**
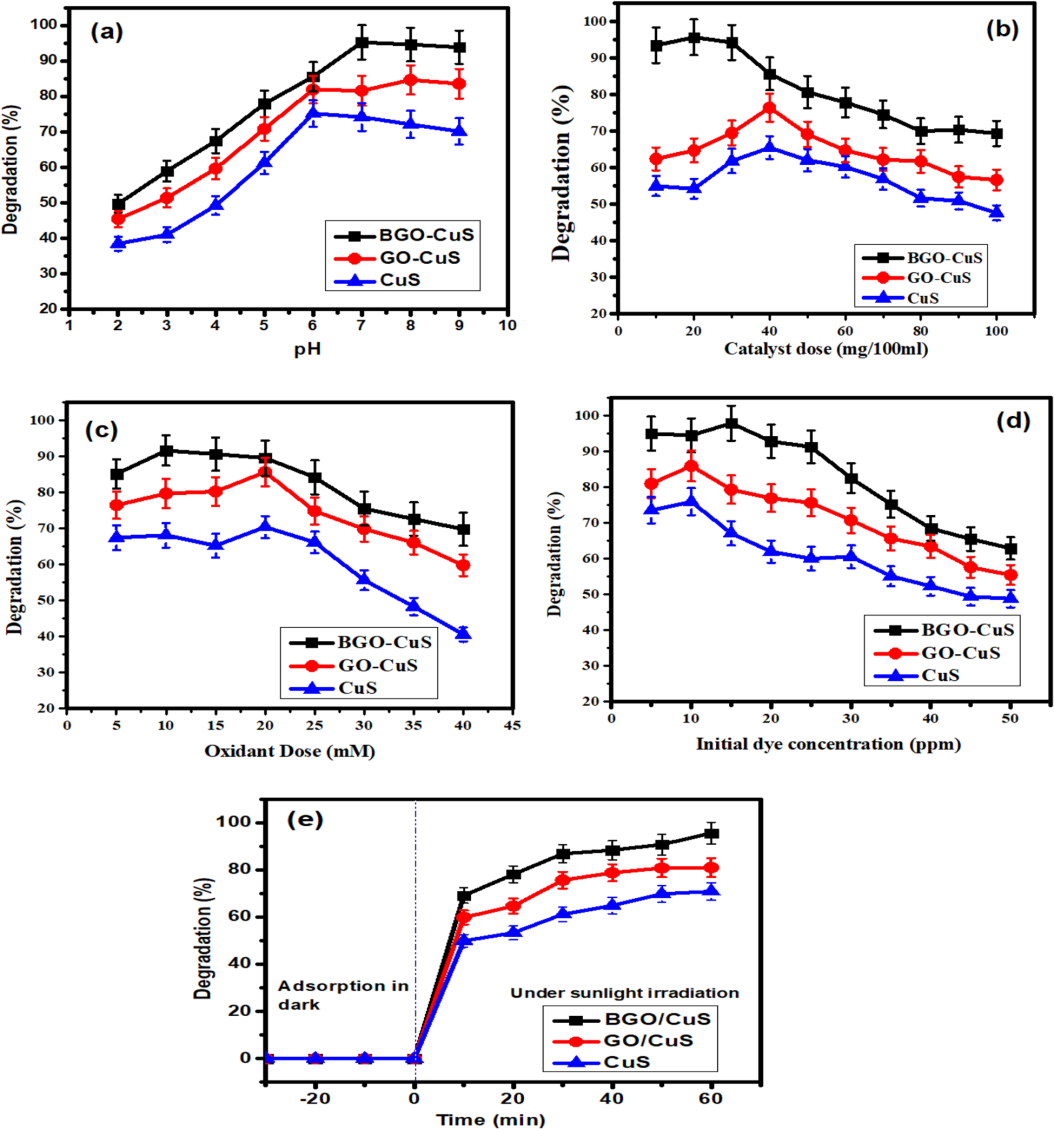
Optimization of reaction parameters using BGO-CuS, GO-CuS, and CuS (**a**) pH, (**b**) catalyst dose, (**c**) oxidant dose, (**d**) Initial dye concentration, (**e**) Irradiation time.

The effect of various operational parameters was assessed for MB degradation by BGO/CuS under sunlight irradiation. A standard MB curve was drawn to optimize initial MB concentration for effective degradation, shown in Fig. S3. The catalyst pH is one of the most crucial criteria in determining how effectively a photocatalyst functions. The photocatalytic activity of CuS/BGO, GO-CuS, and Copper Sulfide was studied at several pH values, ranging from 2 to 9. Figure 8a depicts the results generated by collecting data at predetermined intervals. CuS/BGO exhibited maximum photocatalytic activity at a pH value of 8. At the same time, GO-CuS and CuS showed optimum pH at 8 and 6, respectively. The photocatalytic efficiency increases as the pH rise from acidic to basic. Cationic dyes like methylene blue may be broken down more efficiently when the pH level exceeds the point of zero charge of the catalyst because the catalyst’s surface becomes negatively charged^53^. The pH_PZC_ of BGO-CuS is shown in Fig. S4 of supplementary information. Methylene blue, a cationic dye, dissociates into positively charged molecules and can adhere well to the catalyst's negative charge. At a pH of 8, this catalyst reaches its maximum level of degradation, up to 95%. The increased degradation rate showed that BGO-CuS constructed a layer of heterojunction between BGO and CuS at alkaline pH, leading to charged particles dissociating and generating new active sites. Photocatalytic systems work efficiently at neutral pH for real effluent treatment, which cannot be stressed, mainly when visible light is available^59^.

### Catalyst amount effect on MB degradation

The effectiveness of the photocatalytic process is significantly changed by the amount of catalyst used in the degradation process. BGO-CuS was explored to determine the appropriate amount of the catalyst for the process. An accompanying figure shows the results of this experiment. From 10 mg/100 mL to 100 mg/100 mL, it is apparent that the photocatalytic efficacy of the combination will increase to a particular extent after then, the rate of photodegradation activity would gradually decrease. BGO-CuS showed effective degradation at 20 mg/100 mL catalyst, while GO-CuS and CuS showed degradation at 40 mg/100 mL, respectively, as depicted in graph Fig. 8b. The photocatalytic efficiency is enhanced because of the number of active sites in the photocatalyst. The adsorption of many dye molecules on easily accessible reactive sites accelerates the degradation process. Degradation efficiency can only be improved to a certain point (20 mg/100 mL for BGO-CuS). Photocatalytic performance starts to decline beyond this point. Increased nanoparticle density leads to less surface area and active sites for dye molecules to adsorb, which lowers the efficacy of dye removal by the process. Therefore, a smaller surface area and fewer active sites are accessible for adsorption^60^.

Additionally, dispersion from the surface reduces the quantity of solar energy that reaches the active spots. This means that electrons from VB to CB cannot be excited because of a lack of energy^61^. Degradation of dyes requires a precise balance of catalyst and dye molecules to get optimal results.

### Oxidant dose effect on MB degradation

The oxidant dosage was tuned by varying the quantity of hydrogen peroxide in the dye solution from 5 to 40 mM/100 mL after pH and catalyst concentrations were set constant. After obtaining experimental data at predetermined intervals, a graph was plotted, shown in Fig. 8c. Initially, the experiment was performed without using an oxidant. The catalyst showed only 55% degradation. The photocatalytic effectiveness of the catalyst improved as the oxidant was added, and the concentration of the oxidant was increased to a specific point. Hydroxyl radical (OH^•^) help catalysts in degrading organic dyes more effectively. MB degradation usually occurs after the addition of H_2_O_2_ as it is used to capture the electrons for the generation of hydroxyl radicals. This generation of these OH^**•**^ radicals increases the degradation of dye MB as it gets adsorbed on the catalyst surface. At the same time, more active sites are generated after receiving electrons from the conduction band responsible for OH^**•**^ generation, which is key active species in the degradation process^62^. With an increase in oxidant dosage of 10 mM/100 mL, the photocatalytic activity of BGO-CuS begins to decline. GO-CuS and CuS showed optimum oxidant dose at 20 mM/100 mL. Additionally, hydroxyl radicals play a role in charge separation because they may take electrons from the conduction band. Hydroxyl radicals are formed when hydrogen peroxide absorbs an electron from the superoxide radical.

$${\text{H}}_{{2}} {\text{O}}_{{2}} + {\text{O}}_{{2}} - \to {\text{ HO}} - + {\text{ HO}}* + \,{\text{O}}_{{2}}$$

$${\text{H}}_{{2}} {\text{O}}_{{2}} + {\text{e}} - \left( {{\text{CB}}} \right) \to {\text{HO}} - + {\text{ HO}}^{ \bullet }$$

After a certain limit, the photocatalytic efficiency starts to decrease by further increasing the oxidant dose. This is ascribed to the formation of hydrogen peroxide radicals, which are less reactive than hydroxyl radicals and scavenge them^63^.

$$\normalsize {\text{H}}_{{2}} {\text{O}}_{{2}} + {\text{HO}} \cdot \to {\text{ H}}_{{2}} {\text{O}} + {\text{HO}_{{2}} } \cdot$$

$${{\text{HO}_{{2}} } \cdot} + {\text{HO}} \cdot \to {\text{H}}_{{2}} {\text{O}} + {\text{O}}_{{2}}$$

Hence, the degradation study observed that Boron-doped graphene oxide copper sulfide showed the best photocatalytic performance at 10 mM oxidant concentration.

### Initial dye concentration

Degradation of dye relies significantly on the quantity of dye adsorbed onto the catalyst surface. For the photodegradation of MB by BGO-CuS, GO-CuS, and CuS, the influence of IDC aligning from 5 to 45 ppm was investigated in Fig. 8d. At the same time, all other parameters were kept constant at their optimum levels. An early low MB load led to maximal degradation owing to the highest possible number of available active sites and the reactivity of dye molecules with OH^**•**^ radicals produced on the surface of the composite. More dye molecules adsorb to the catalyst surface as the dye concentration rises, thus, the active sites become saturated. Reduced light intensity at the catalyst surface results in less hydroxyl radical production, resulting in less efficient breakdown of methylene blue molecules^42^. A concentration of 15 ppm was found to be the optimum level for BGO-CuS, and 10 ppm was found to be optimum for GO-CuS and CuS. Even at higher MB concentrations, the BGO-CuS nanocomposite exhibited over 95% MB removal. New dye molecules quickly replace dye molecules degraded on the photocatalyst's surface in the vicinity. Because of this, the unique BGO-CuS photocatalyst is more effective in degrading MB than previous photocatalysts, even when the dye load increases.

### Irradiation time

The irradiation period was the most critical factor in determining the photocatalyst's degrading activities. For time optimization, the oxidant dosage, catalyst concentration, initial dye concentration, and pH were all held constant. In processes similar to those of Fenton, the rate of degradation gradually slows down over time because the pH solution has affected the generation of hydroxyl radicals^64^.

As a result of the interaction of hydroxyl ions with copper, sludge is generated, which inhibits the active site of the catalyst, which in turn causes the Fenton-like reaction to become repressed^65^. This experiment was conducted for BGO-CuS, GO-CuS, and CuS. After 60 min, the BGO-CuS photocatalyst showed 95%, while GO-CuS and CuS showed 79% and 59% degradation, respectively Fig. 8e^66^.

### Kinetics of photodegradation reaction

The kinetics of MB degradation was analyzed by using first and second-order kinetic models for the experimental data. The equations for these models are given below^67^. A linear relation between “ln C_o_/C_t_” and “t” is evident in the figure. The rate constant value for the first-order reaction is calculated from the slope of the graph. Similarly, the plot between “1/C_t_ − 1/C_o_” and time also results in a linear relation, and the slope of this plot gives the value of K2, the rate constant for the second-order reaction. The values for rate constants and respective R^2^ are listed in Table 1. It can be observed from the table that the first-order kinetics model is best fitted for the photocatalytic degradation process as compared to the second-order, as this model shows a higher value for R^2^.

4 $${\text{ln C}}_{{\text{o}}} /{\text{C}}_{{\text{t}}} = {\text{K1t}} \quad \left( {\text{First order}} \right)$$

5 $${1}/{\text{C}}_{{\text{t}}} {-}{1}/{\text{C}}_{{\text{o}}} = {\text{K2t}}\quad \left( {\text{Second order}} \right)$$

**Table 1:** Calculated rate constants for first-order and second-order kinetics for MB degradation by BGO-CuS.

Photocatalyst	K1 (min^−1^) 1st order	R^2^ value for pseudo-first-order	K2 (Lµmol^−1^ min^−1^) 2nd order	R^2^ value for second order
CuS	0.014	0.8687	0.0002	0.7345
GO-CuS	0.0275	0.9088	0.0007	0.8283
BGO-CuS	0.0608	0.9650	0.0051	0.6224

**Figure 9:**
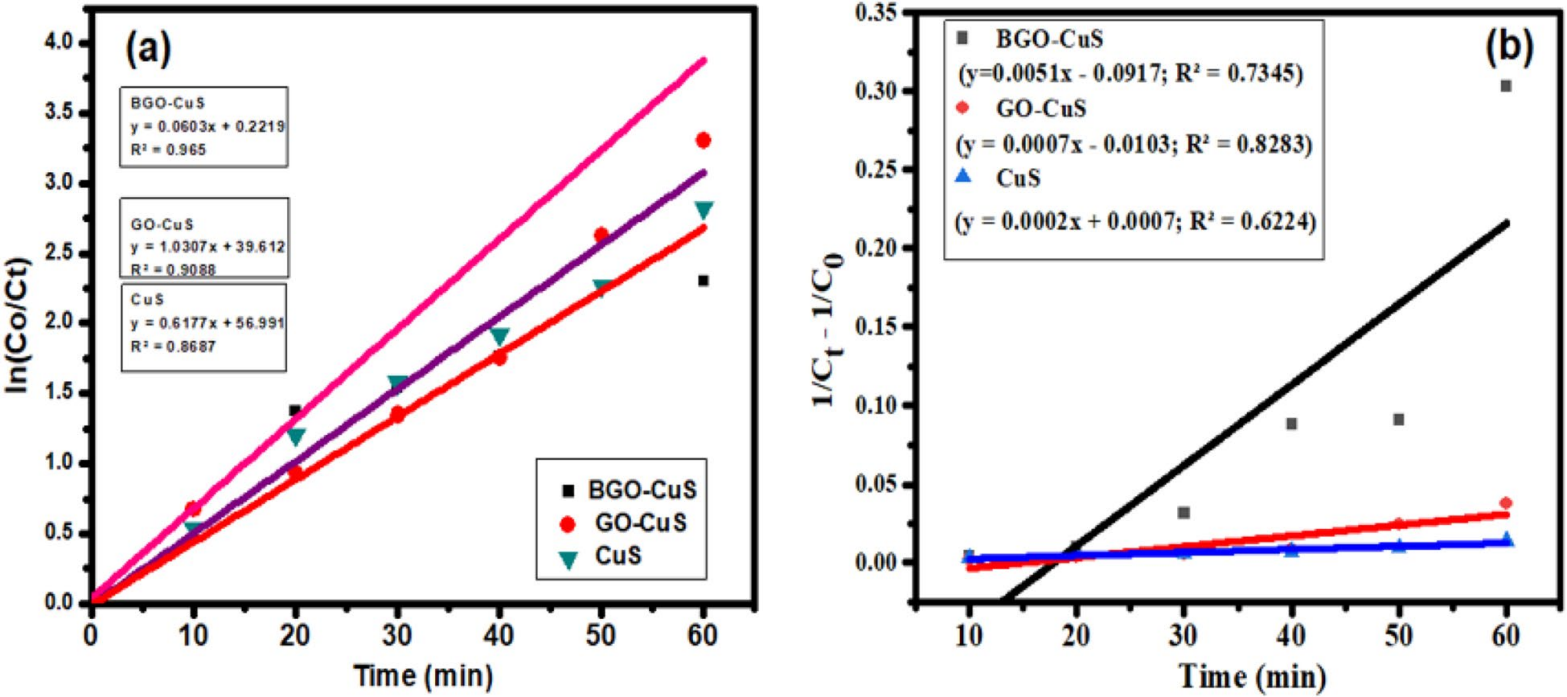
Kinetics of reaction (**a**) pseudo-first-order, (**b**) second-order kinetic model of BGO-CuS, GO-CuS, and CuS.

Here, “C_o_” represents the initial concentration of the dye solution at time zero and “C_t_” at the specific time denoted by “t”^68^. “K1” and “K2” are the rate constant of first and second-order reactions, respectively Fig. 9a, b.

The plot of time vs ln (C_t_/C_o_) yielded a linear result. The k value was analyzed using a pseudo-first-order slope. In BGO-CuS, the doping of Boron into graphene Oxide increased degradation activity as measured by a higher k rate constant (k) value compared to GO-CuS (Table 1).

### Reusability study

**Figure 10:**
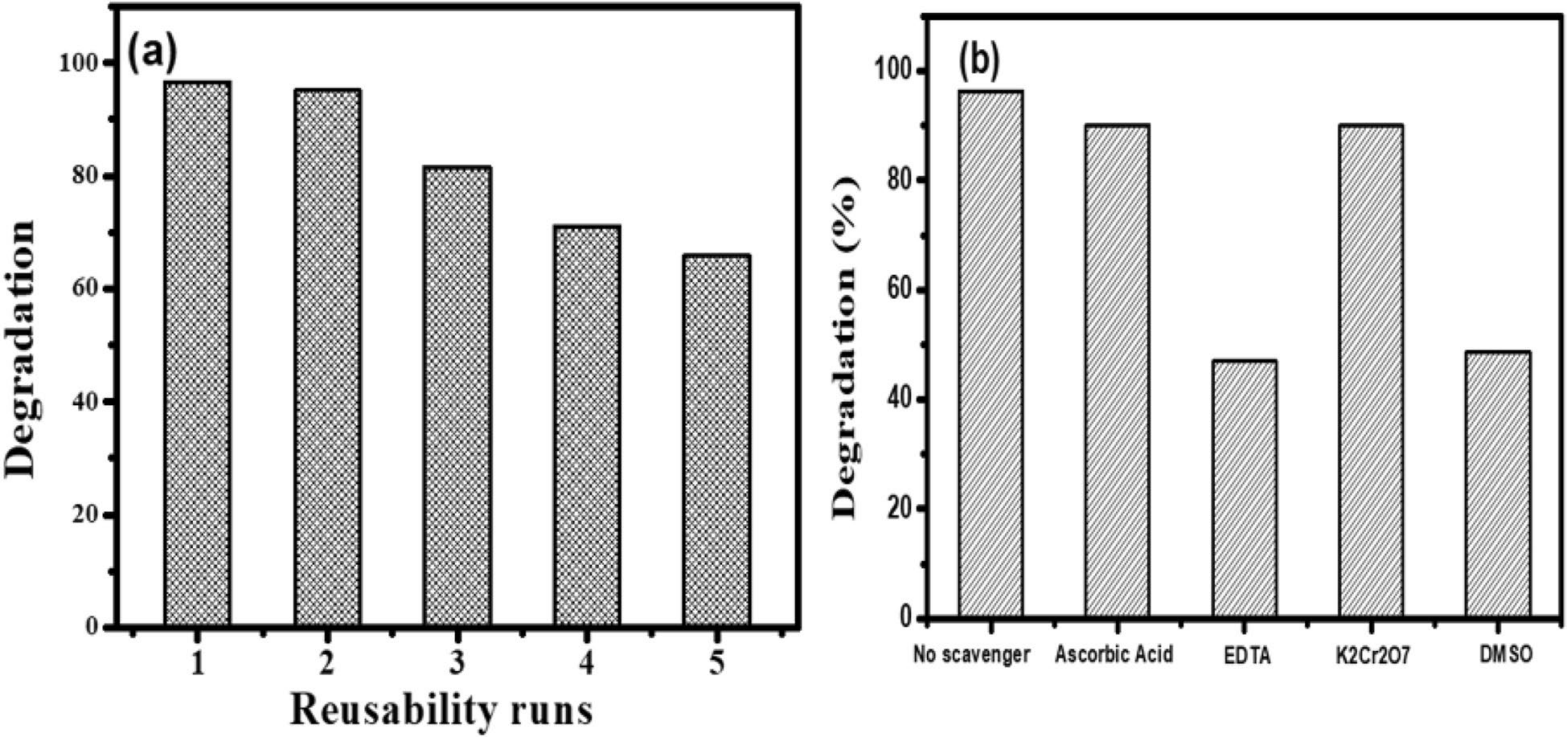
Study of (**a**) reusability study (**b**) various radical scavengers test for BGO-CuS nanocomposite.

The photocatalysts must be stable enough to be reused several times before they can be used in real-world applications. The photocatalytic stability of the BGO-CuS catalyst for MB was evaluated by cycling the catalysts through five consecutive experiments under constant circumstances. Figure 10a shows MB degradation after five cycles. To further broaden the usage of CuS/BGO catalysts in the field of photocatalysis, it was clear from the findings that the as-obtained catalysts had high stability and recycling capacities. The sample was reused after the washing and drying process. The efficiency decreases from 95 to 92%, 85%, 72%, and 65% of BGO-CuS after five successive runs^64^. The XRD of the recovered BGO-CuS sample showed no phase change suggesting stability in the structure of the composite Fig. S6. This explains that more pollutants can be removed with a minimal amount of catalyst used^69^. A comparison of B-doped GO-based nanocomposites for effectively removing organic pollutants has been shown in Table S1 of supplementary information.

The criteria for the efficient photocatalytic performance of a catalyst is the low leaching of metal ions during the degradation studies. In five cycles, the effect of potentially leached Cu ions was assessed using atomic absorption spectroscopy (AAS). The AAS analysis showed minimal copper metal ions leaching at acidic and basic conditions. Less than 1% loss of Cu ions was observed, which is very minimal (i.e. 0.1 mg on average per reusability trial) owing to diverse factors like reusability of the catalyst, including its separation and rinsing each time for the next reuse. These values in any case, should not exceed 2.0 mg/L according to European directives^67^. Hence, the leaching values of Cu ions should be below these values. The decline in catalytic ability in the reusability trial is hence not owing to leaching but the blocking of active sites due to the adsorption of more pollutant molecules^66^.

The degree of mineralization of MB by BGO-CuS nanocatalyst was determined using the Total organic content (TOC). Figure S5a shows a plot of absorbance against the wavelength of MB at various time intervals. This graph shows a visible decline in intensity with 95% degradation over 60 min. Figure S5b shows the TOC values for the MB solution at various time intervals. The TOC values decreased up to 86% under sunlight irradiation. Hence, BGO-CuS showed improved degradation efficiency of MB dye molecules, converting organic carbon mostly into carbon dioxide during the process.

### Scavenging test and Proposed mechanism of MB degradation by BGO-CuS

The reactive species such as holes (h^+^), electrons (e^−^), and hydroxyl (OH^**•**^) radicals are involved in photocatalytic degradation. So, the efficiency of radicals responsible for degradation can be checked by using radical scavenging species. Radical scavengers such as Ethylene diamine tetra-acetate for holes, Potassium dichromate (K_2_Cr_2_O_7_) for electrons, and dimethyl sulfoxide for hydroxide radicals are used as scavenging agents against hydroxyl radicals. 5 mM of each scavenger was used under sunlight at optimum conditions. The result shows that the degradation of the dye solution was drastically reduced by using EDTA and DMSO. The degradation was decreased from 95 to 37% by using EDTA and 65% by using DMSO for BGO/CuS, suggesting holes and hydroxyl radicals are key species in degradation Fig. 10b. Similarly, the contribution of electrons and hole scavengers also reduced MB degradation.

The hypothesized mechanism for the dye photodegradation is based on holes and hydroxyl radicals, which both play significant roles in the process. According to what we know, the holes and electrons involved in the degradation process are formed when more light energy is absorbed than bandgap electrons and holes are responsible for the initiation of the degradation process and are produced as soon as the composite surface is irradiated energy is absorbed. According to the previously published reports, the conduction band potential and valence band edge potential of CuS are − 0.28 eV and + 1.83 eV, respectively. Due to a negative CB potential electrons will move from VB of CuS to CB of CuS, and holes will be formed in VB of CuS. From here, electrons will move from CB of CuS to VB of BGO.

**Figure 11:**
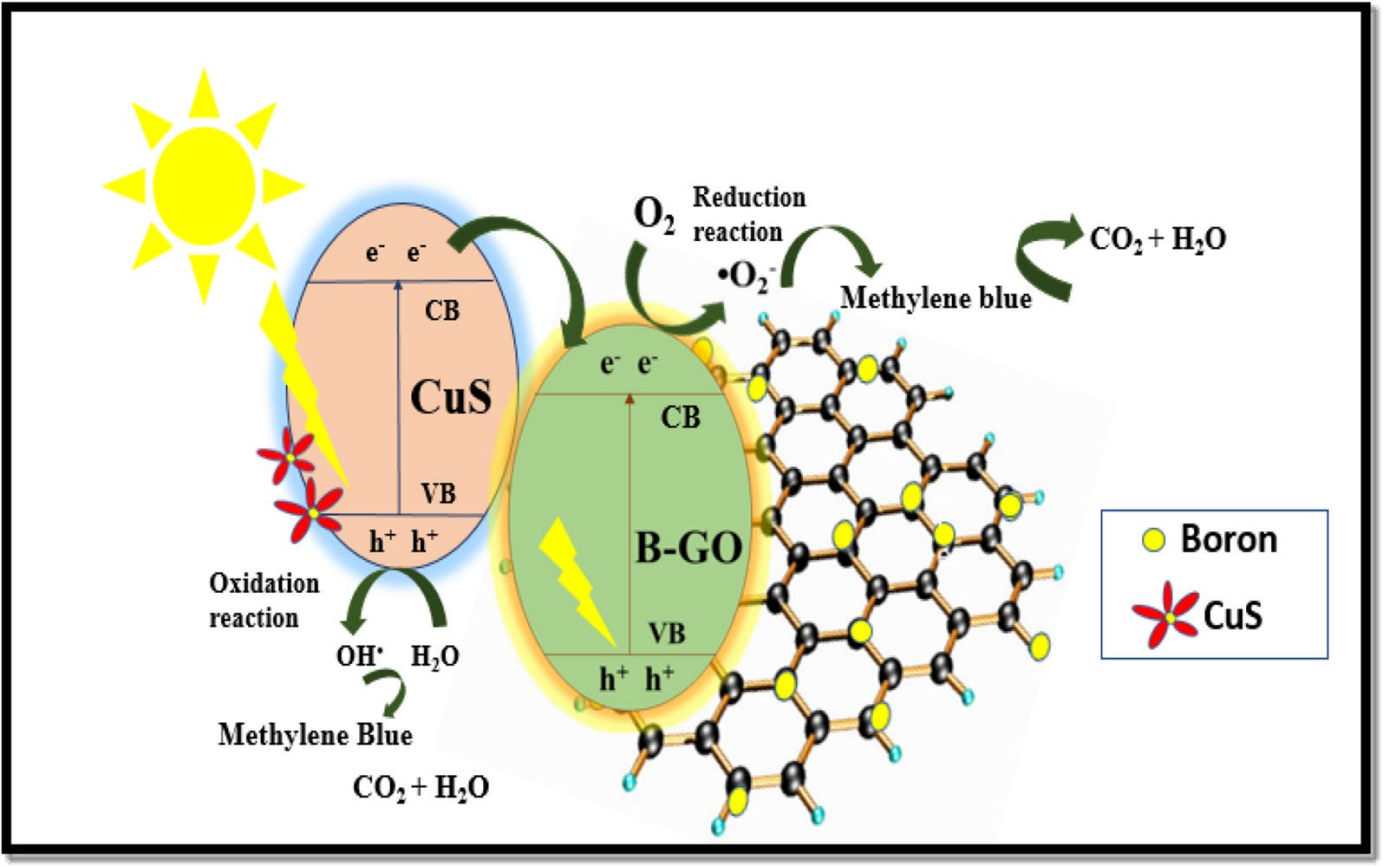
Proposed mechanism of dye methylene blue degradation by BGO-CuS.

Due to the doping of boron in GO, which lowers the bandgap of CuS by changing energy level as a result of coupling and easy charge transfer, the electrons are readily stimulated from the valence band toward the conduction band when sunlight strikes the surface of BGO-CuS. These photo-excited electrons travel away from the VB of copper Sulfide and towards CB of boron-GO, while the h^+^ atoms stay in the VB. In graphene, oxide holes shift closer to the VB of CuS, increasing the amount of time it takes for recombination. The H_2_O_2_ was reduced by these photo-generated holes, resulting in superoxide radical production. The Sp2 structure of doped GO additionally provides many delocalized electrons improving light-generated charge carrier movement. Consequently, these electrons and adsorbed oxygen generate superoxide anion radicals in rGO sheets, while holes and water molecules react and form hydroxyl radicals over the surface of CuS/ BGO. The proposed mechanism is as following shown in Fig. 11.

$${\text{CuS}}/{\text{BGO}} + hv \to {\text{CuS}}\left( {{\text{e}}^{ - } + {\text{ h}}^{ + } } \right)/{\text{BGO}}\left( {{\text{e}}^{ - } + {\text{ h}}^{ + } } \right)$$

$${\text{CuS}}\left( {{\text{e}}^{ - } + {\text{ h}}^{ + } } \right)/{\text{BGO}}\left( {{\text{e}}^{ - } + {\text{ h}}^{ + } } \right) \to {\text{CuS}}\left( {{\text{h}}^{ + } } \right)/{\text{BGO}}\left( {{\text{e}}^{ - } } \right)$$

$${\text{BGO}}\left( {{\text{e}}^{ - } } \right) + {\text{O}}_{{2}} \to {\text{O}}_{{2}}^{. - }$$

$${\text{CuS}}\left( {{\text{h}}^{ + } } \right) + {\text{OH}}^{ - } \to {\text{CuS}} + {\text{OH}}^{ \bullet }$$

$${\text{O}}_{{2}}^{. - } /{\text{OH}}^{ \bullet } + {\text{MB}} \to {\text{Intermediates}} \to {\text{Degraded}}\;{\text{Products}}$$

## Response surface methodology

The response surface methodology (RSM) is a unique approach for optimizing several elements that participated in the photocatalytic degradation process. Within the scope of this investigation, the central composite design methodology was used to investigate how various characteristics influenced the rate at which methylene blue was degraded. The use of CCD allowed for the management of independent factors, i.e. pH, time, and catalyst dose to achieve the highest possible percentage of degradation (the value of the response variable).

### Analysis of variance (ANOVA)

The fit summary plot makes it very clear that the applied model greatly impacts the optimized variables. The high R^2^ value also shows the significant predictive power of the model (Table 2). When a quadratic model was used, it was discovered that there is a robust connection between factors such as pH, time, catalyst concentration, and the amount of Degradation that has occurred. A second-order polynomial equation (eq. 6) was used to analyze the link between the aforementioned factors and the proportion of methylene blue dye that was degraded. Here, Y denotes the response of the dependent variable (percentage degradation), β° represents a coefficient having a specific value, β_i_ is a linear coefficient, and the quadratic coefficient is denoted by β_ii_. In contrast, the coefficient value for interaction effects is represented by β_ij_. Moreover, coded values ﻿for independent variables are represented by x_i_ and x_j_ while ∈ stands for random errors.

6 $${\text{Y}} = {\upbeta }_{^\circ } + \mathop \sum \limits_{{{\text{i}} = 1}}^{{\text{k}}} {\upbeta }_{{\text{i}}} {\text{x}}_{{\text{i}}} + { }\mathop \sum \limits_{{{\text{i}} = 1}}^{{\text{k}}} {\upbeta }_{{{\text{ii}}}} {\text{x}}_{{\text{i}}}^{2} + \mathop \sum \limits_{{{\text{i}} = 1}}^{{\text{k}}} \mathop \sum \limits_{{{\text{i}} \ne {\text{j}} = 1}}^{{\text{k}}} {\upbeta }_{{{\text{ij}}}} {\text{x}}_{{\text{i}}} {\text{x}}_{{\text{j}}} + \in$$

**Table 2:** ANOVA table for MB degradation by novel BGO-CuS.

Source	Sum of squares	df	Mean square	F-vaue	*p* value	
Model	10,582.64	9	1175.85	132.52	< 0.0001	Significant
A-pH	943.10	1	943.10	106.29	< 0.0001	
B-catalyst dose	167.64	1	167.64	18.89	0.0015	
C-Time	1340.11	1	1340.11	151.03	< 0.0001	
AB	185.57	1	185.57	20.91	0.0010	
AC	424.42	1	424.42	47.83	< 0.0001	
BC	884.73	1	884.73	99.71	< 0.0001	
A^2^	3912.12	1	3912.12	440.91	< 0.0001	
B^2^	501.28	1	501.28	56.50	< 0.0001	
C^2^	3229.57	1	3229.57	363.98	< 0.0001	
Residual	88.73	10	8.87			
Lack of fit	37.42	5	7.48	0.7293	0.6313	Not significant
Pure error	51.31	5	10.26			
Cor. total	10,671.37	19				
Std. dev.	2.98		R^2^	0.9917		
Mean	69.97		Adjusted R^2^	0.9842		
C.V. %	4.26		Predicted R^2^	0.9661		
			Model precision	38.7791		

Regression analysis and lack of fit decide the model fit for particular information. The P-value tells us about the significant or non-significant variables. If *p* > 0.1000, the model becomes non-significant, while *p* < 0.05 shows a significant result. The model is highly significant if the *p* value is less than 0.0001.

7 $$\begin{gathered} {\text{Y}} = {95}.{4735} + {8}.{31}00{6}*{\text{A}} + - {3}.{5}0{359}*{\text{B}} + {9}.{9}0{591}*{\text{C}} + {4}.{81625}*{\text{AB}} + \hfill \\ \quad \;\; - {7}.{28375}*{\text{AC}} + {1}0.{5162}*{\text{BC}} + - {16}.{4761}*{\text{A}}^{{2}} + - {5}.{89779}*{\text{B}}^{{2}} + - {14}.{97}*{\text{C}}^{{2}} \hfill \\ \end{gathered}$$

Here in eq. 7, Y represents the percentage degradation of methylene blue dye, while A, B, and C stand for pH, catalyst concentration, and oxidant dose, respectively. AB, AC, and BC depict the linear correlation between independent variables, while A^2^, B^2^, and C^2^ represent quadratic effects.

**Figure 12:**
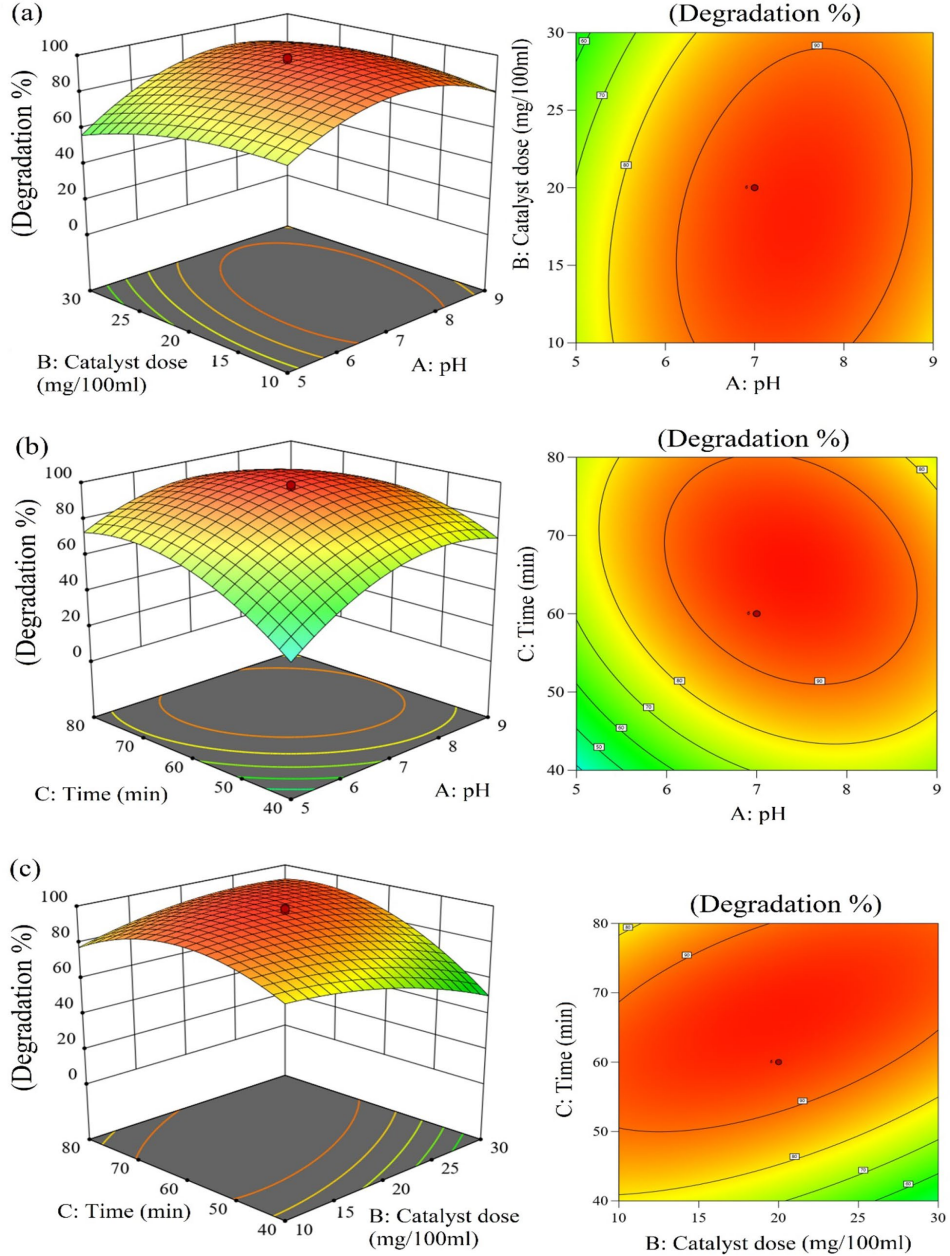
3D and contour plots of (**a**) pH and catalysts conc., (**b**) pH and time, (**c**) time and catalyst conc.

The effects of changing both the pH and the amount of catalyst are shown in Fig. 12a. This relationship between the variables is established on both a contour plot and a three-dimensional display. The study was conducted in a pH range of 5–9 with a catalyst concentration of 10–30 mg/100 mL. If the pH is raised from 5 to 9, the degradation rate rises, showing maximum Degradation at 7 pH, then drops. The photocatalytic Degradation of methylene blue also improved between 10 and 30 mg/100 mL of catalyst concentration. The larger photocatalyst surface area is responsible for this improvement because it offers more dye adsorption sites. With further increases in catalyst concentration, nanoparticle aggregation and subsequent blocking of active sites occur. Consequently, the rate at which a photocatalyst degrades an object when exposed to sunlight decreases.pH and time interaction can be seen in Fig. 12b. Time in the range of 40 to 80 min was optimized for maximum MB degradation. Increasing the time from 40 to 80 min improved photocatalytic performance. After 60 min, maximum Degradation was obtained after that no significant changes were observed.

Catalyst concentrations between 10 and 30 mg/100 mL and time between 40 and 80 min were used in the studies. The degradation rate was maximized at 20 mg/100 mL and 60 min. In Fig. 12c their mutual interaction is graphically shown.

## Conclusion

The unique composite BGO-CuS has shown effective degradation for the removal of the dye methylene blue in solar light. An ultrasonication-assisted hydrothermal synthesis method was used to synthesize the doped BGO-CuS. Under sunlight, the composite demonstrated remarkable efficacy in degrading the methylene blue dye. Additionally, undoped GO-CuS and CuS were made to make a comparison of degradation efficiencies among undoped binary and pristine components. In only one hour, BGO-CuS demonstrated enhanced removal of MB dye up to 95%. The morphology and crystal structure of the novel nanohybrid was evaluated using various characterization techniques, including SEM–EDS, FTIR, XRD, XPS, BET, PL, and UV–Vis DRS spectroscopy. The heterojunction created as a consequence of the combination of copper sulfide and GO and doping boron in GO was attributed to being responsible for the increased photocatalytic efficiency. Five consecutive cycles were performed with a minimal loss of a catalyst and effective degradation up to the 5th cycle, to test the photostability of the photocatalyst.

## Supplementary Information

Supplementary Information.

## Data Availability

All raw data generated or analysed during this study are included in this published article as supplementary material. Any other information is available from the corresponding author on reasonable request.
