# Supplementary material for: Investigation of boron-doped graphene oxide anchored with copper sulphide flowers as visible light active photocatalyst for methylene blue degradation
Source: Sci Rep. 2023 Jun 12;13:9497. doi: 10.1038/s41598-023-36486-6 (PMC10261073; doi:10.1038/s41598-023-36486-6)
Supplement: Supplementary file 1 — Supplementary Information. [file 41598_2023_36486_MOESM1_ESM.docx]

**Investigation of boron-doped graphene oxide anchored with copper sulphide flowers as visible light active photocatalyst for methylene blue degradation**

Ahmad Farhan^a^, Muhammad Zahid*****^a^**,** Noor Tahir^a^, Asim Mansha^b^, Muhammad Yaseen^c^, Ghulam Mustafa^d^, Mohammed A. Alamir^e^, Ibrahim M. Alarifi^f^ , Imran shahid*^g^

1. **Synthesis**

**1.1 Synthesis of Graphene Oxide:**

Graphene Oxide was produced by modified Hummer's method. Initially, 5g of Graphitic powder and 2.5g of sodium nitrate was weighed on the weighing balance.150ml of 98% H_2_SO_4_ was taken in conical flask and mechanically stirred. 150 ml of 98% H_2_SO_4_ was added in above solution dropwise under constant mechanical stirring. 30g of KMnO_4_ was added to the solution pinch by pinch under ice bath to start the oxidation process. The resulting solution was mechanically stirred until brown color solution was obtained. The temperature was maintained at 37-39^o^C during that process. After that 200-240 ml of distilled water was added into the brown solution with continues mechanical stirring. This reaction was highly exothermic, and temperature was maintained at 85-95 ^o^C in an oil bath. After reaction was complete, 30 ml of H_2_O_2_ was added to the above solution. The solution turned yellow. This mixture was placed until brown suspension of GO was formed. Neutral pH for GO was obtained by washing it many times with distilled water and centrifuged. The obtained product was ultrasonicated for 20 minutes followed by drying at 80 oC for 12 hours to get the thin film of GO [1].


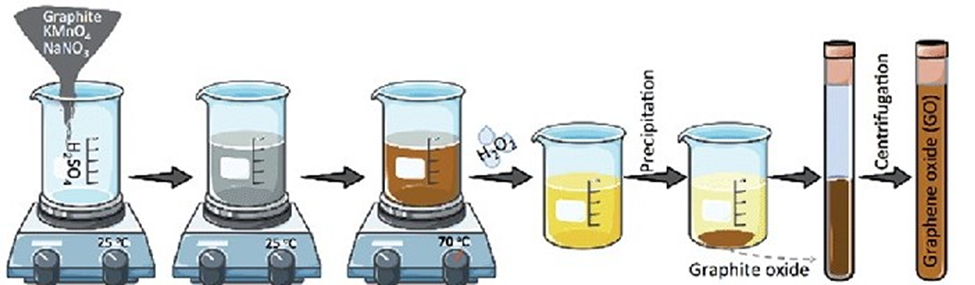


**Figure S1. Schematic diagram of Preparation of graphene oxide**

- 1. **Synthesis of BGO**

Graphene Oxide was synthesised using a modified Hummer's technique. Boron-doped GO was made by combining GO powder with various concentrations of boric acid in an aqueous solution. For 6 hours, the mixture was vacuum dried. It was then heated to 700 degrees Celsius at a rate of 5 degrees Celsius per minute. It was then pyrolyzed for 2 hours under nitrogen flow. The product was washed with ethyl alcohol and Deionized water to remove residual ions. The finished product was dried in a 60^o^C oven for 12 hours [2].

**1.3 Synthesis of GO/CuS**

The hydrothermal technique/method was used to make the CuS/GO nanocomposites. 1 mmol copper nitrate (Cu(NO_3_)_2_ was added to 40 mL DI water, and the solution was constantly agitated. 2.5 mmol thiourea was added as soon as a blue solution had formed. After the solution had been well mixed, 0.02g of GO was added to the mixture and autoclaved for 24 hours at 150°C. CuS/GO nanocomposite was made by centrifuging the black precipitate with distilled ethanol, water, and keeping it at room temperature [3].

**2. Characterization**

**2.1 EDS elemental mapping**

The elemental mapping images reveal the presence of various prominent elements in respective weight percentages on the surface of nanocomposite in **Fig.S2**


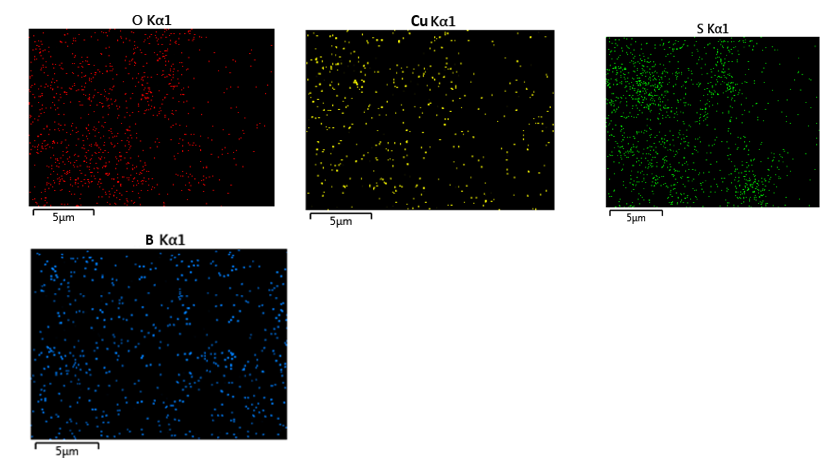


**Figure S2. Elemental mapping images of various elements in BGO-CuS**

3. **Calibration curve**

The absorbance of the dye solution can be measured using a spectrophotometer set to a wavelength of 664 nm, which will allow one to generate a calibration curve. For the purpose of creating a standard curve, dye solutions of varying concentrations, ranging from 2 ppm to 12 ppm, were used. When more dye is added to a solution, it causes the solution to have a higher absorbance. The value of R2 provides information on the accuracy of the graph.


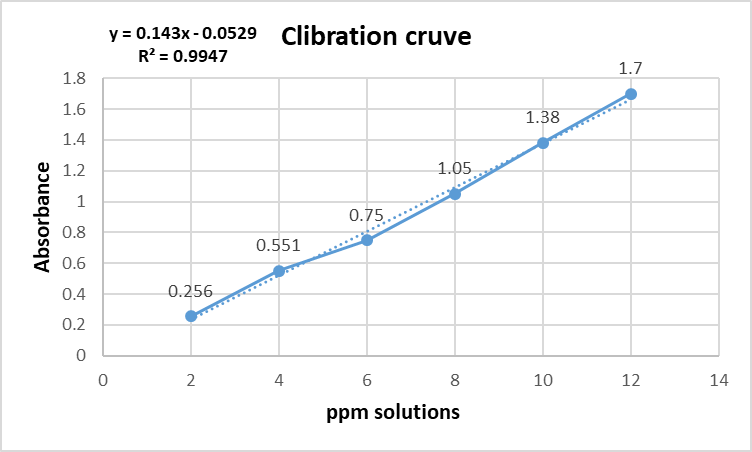


**Figure S3. Standard calibration curve**

# Point Zero Charge

A solution of 0.01 M sodium chloride was made in order to determine the point zero charge. On a hotplate, the solution that had been made was put so that CO_2_ could be removed. Following the appearance of bubbles, the beaker that held the solution was taken off of the hotplate and set aside. The pH of the NaCl solution was adjusted to the values of 2, 4, 6, 8, and 10 with the help of a 0.1M solution of HCL, as well as NaOH. After that, composite in the ratio of 75 mg/50 ml was added to each beaker after the pH had been adjusted. After covering the beakers with aluminum foil, they were put on an orbital shaker in the ultraviolet chamber for a period of twenty-four hours. The ultimate pH was determined after 24 hours had passed. The surface charge of the composite was found to be proportional to the difference between the initial and final pH levels. Results depicted the point zero charge on BGO-CuS was 5.0 pH [4].


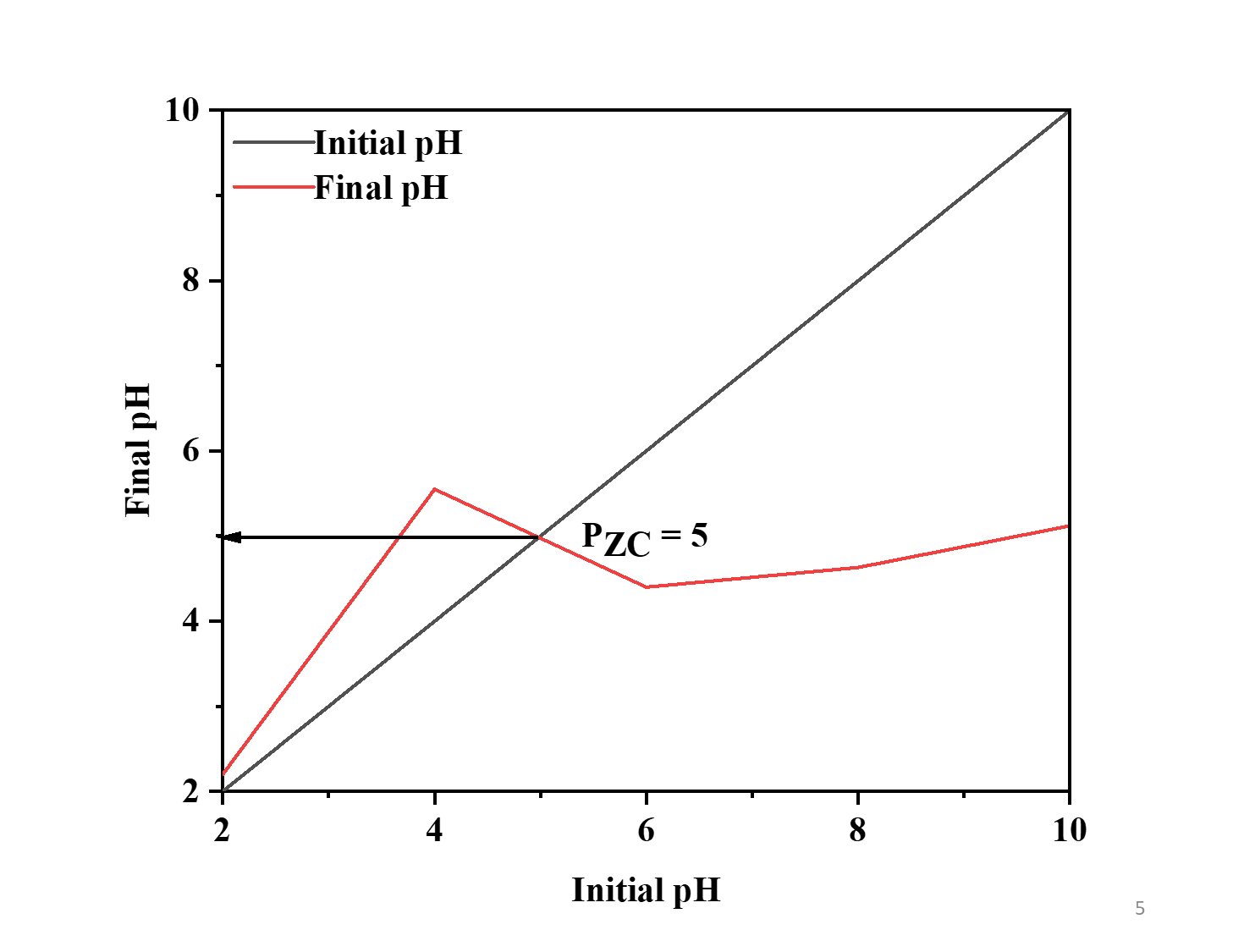


**Figure S4. Point zero charge of boron doped graphene oxide copper oxide**


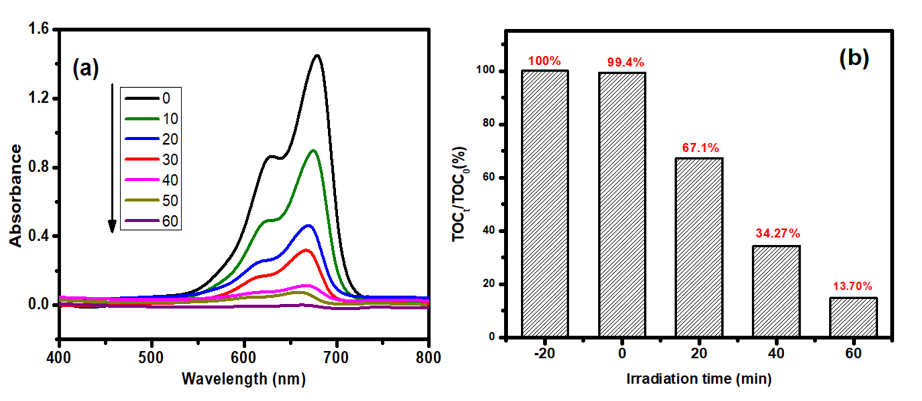


**Figure S5. (a) UV-Visible Scans of MB degradation by BGO-CuS with time (b) Total organic content estimation**


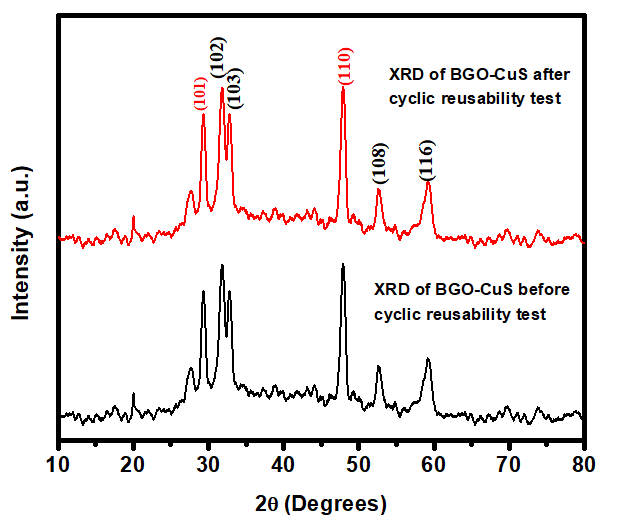


**Figure S6. XRD of treated BGO-CuS sample**

**Table S1. Comparison between various copper sulfide-based heterostructures for degradation of various pollutants.**

| **Composites** | **Fabrication method** | **Model**  **Polutant** | **Catalyst dose (mg/l)** | **pH** | **Efficiency**  **(%)** | **Time**  **(min)** | **Reference** |
| --- | --- | --- | --- | --- | --- | --- | --- |
| Bi_2_WO_6_/CuS | Solvothermal method | Organic  Pollutants and cr(vi) | - | 2-6 | 74.7 and 75.7 | 105 | [[59](#_ENREF_59)] |
| CoFe_2_O_4_@CuS | Heat treatment | Penicillin G antibiotic | 200 | 5 | 70.7 | 120 | [[60](#_ENREF_60)] |
| KCC-1/CuS | Chemical bath deposition | Humic acid | 100 | 3 | 89.5 | 90 | [[61](#_ENREF_61)] |
| polyaniline/copper sulfide | In situ precipitation  Method | Ulfamethoxazole | 40 | 2 | 72.13 | - | [[62](#_ENREF_62)] |
| BiOCl/CuS | Hydrothermal  Method | Rhodamine b | - | - | 96 | 100 | [[63](#_ENREF_63)] |
| Ag-doped CuS | Hydrothermal  Method | Organic pollutants. | - | - | 93.8 | 30 | [[64](#_ENREF_64)] |
| rGO/CuS | In-situ reduction | Malachite green dye | 500 | 5 | 97.6 | 90 | [[66](#_ENREF_66)] |
| CuS/Bi_2_O_2_CO_3_ | Hydrothermal  Method | Chlorpyrifos | 25 | 4 | 95.6 | 180 | [[67](#_ENREF_67)] |
| CuS@carbon | In situ | 2,4-dichlorphenol | - | 3 | 90 | 150 | [[68](#_ENREF_68)] |
| CuS/CuO/Cu | Liquid–solid reaction | Methylene blue | - | - | 98.7 | 40 | [[69](#_ENREF_69)] |
| TiO2/rGO/CuS | Electrochemical anodization | Rhodamine b | 10 | - | 100 | 110 | [[47](#_ENREF_47)] |
| flower-like CuS/RGO | solvothermal method, | Methylene blue | 200 | - | 100 | 140 | [[70](#_ENREF_70)] |
| CuS/g-C3N4 | In-situ Synthesis | Methylene blue | 330 | 4.5 | 98 | 120 | [[71](#_ENREF_71)] |
| BGO/CuS | Hydrothermal method | Methylene blue | 100 | 7 | 96.7 | 60 | This Work |

1. N. Tahir, M. Zahid, I.A. Bhatti, and Y. Jamil, Fabrication of visible light active Mn-doped Bi2WO6-GO/MoS2 heterostructure for enhanced photocatalytic degradation of methylene blue, Environ. Sci. Pollut. R. 29 **(**2022) 6552-6567.

2. H. Yang, A. Züttel, S. Kim, Y. Ko, and W. Kim, Effect of boron doping on graphene oxide for ammonia adsorption, ChemNanoMat 3 **(**2017) 794-797.

3. M. Saranya, R. Ramachandran, P. Kollu, S.K. Jeong, and A.N. Grace, A template-free facile approach for the synthesis of CuS–rGO nanocomposites towards enhanced photocatalytic reduction of organic contaminants and textile effluents, RSC Advances 5 **(**2015) 15831-15840.

4. A.H. Zyoud, A. Zubi, S.H. Zyoud, M.H. Hilal, S. Zyoud, N. Qamhieh, A. Hajamohideen, and H.S. Hilal, Kaolin-supported ZnO nanoparticle catalysts in self-sensitized tetracycline photodegradation: zero-point charge and pH effects, Applied Clay Science 182 **(**2019) 105294.

5. W. Mao, L. Zhang, T. Wang, Y. Bai, and Y. Guan, Fabrication of highly efficient Bi2WO6/CuS composite for visible-light photocatalytic removal of organic pollutants and Cr (VI) from wastewater, Front. Environ. Sci. Eng. 15 **(**2021) 1-13.

6. M. Kamranifar, A. Allahresani, and A. Naghizadeh, Synthesis and characterizations of a novel CoFe2O4@ CuS magnetic nanocomposite and investigation of its efficiency for photocatalytic degradation of penicillin G antibiotic in simulated wastewater, J. Hazard. Mater. 366 **(**2019) 545-555.

7. N. Mohammadi, A. Allahresani, and A. Naghizadeh, Enhanced photo-catalytic degradation of natural organic matters (NOMs) with a novel fibrous silica-copper sulfide nanocomposite (KCC1-CuS), J. Mol. Struct. 1249 **(**2022) 131624.

8. J. Yang, L. Fang, X. Gan, G. Meng, H. Li, and Y. Jia, Efficient degradation of sulfamethoxazole under visible light irradiation by polyaniline/copper sulfide composite photocatalyst, Environ. Sci. Pollut. R. **(**2022) 1-10.

9. X. Wang, H. Hu, S. Chen, K. Zhang, J. Zhang, W. Zou, and R. Wang, One-step fabrication of BiOCl/CuS heterojunction photocatalysts with enhanced visible-light responsive activity, Mater. Chem. Phys. 158 **(**2015) 67-73.

10. R. Wang, G. Shan, T. Wang, D. Yin, and Y. Chen, Photothermal enhanced photocatalytic activity based on Ag-doped CuS nanocomposites, J. Alloys Compd. 864 **(**2021) 158591.

11. S. Harish, J. Archana, M. Navaneethan, S. Ponnusamy, A. Singh, V. Gupta, D. Aswal, H. Ikeda, and Y. Hayakawa, Synergetic effect of CuS@ ZnS nanostructures on photocatalytic degradation of organic pollutant under visible light irradiation, RSC advances 7 **(**2017) 34366-34375.

12. S.I. El-Hout, S.M. El-Sheikh, A. Gaber, A. Shawky, and A.I. Ahmed, Highly efficient sunlight-driven photocatalytic degradation of malachite green dye over reduced graphene oxide-supported CuS nanoparticles, J. Alloys Compd. 849 **(**2020) 156573.

13. D. Majhi, Y. Bhoi, P.K. Samal, and B. Mishra, Morphology controlled synthesis and photocatalytic study of novel CuS-Bi2O2CO3 heterojunction system for chlorpyrifos degradation under visible light illumination, Appl. Surf. Sci. 455 **(**2018) 891-902.

14. Y. Chen, R. Su, F. Wang, W. Zhou, B. Gao, Q. Yue, and Q. Li, In-situ synthesis of CuS@ carbon nanocomposites and application in enhanced photo-fenton degradation of 2, 4-DCP, Chemosphere 270 **(**2021) 129295.

15. C. Wu, Y. Sun, Z. Cui, F. Song, and J. Wang, Fabrication of CuS/CuO nanowire heterostructures on copper mesh with improved visible light photocatalytic properties, J. Phys. Chem. Solids 140 **(**2020) 109355.

16. R.M. Gunnagol and M.H.K. Rabinal, TiO2/rGO/CuS nanocomposites for efficient photocatalytic degradation of Rhodamine‐B dye, ChemistrySelect 4 **(**2019) 6167-6176.

17. X.-S. Hu, Y. Shen, Y.-T. Zhang, H.-F. Zhang, L.-H. Xu, and Y.-J. Xing, Synthesis of flower-like CuS/reduced graphene oxide (RGO) composites with significantly enhanced photocatalytic performance, J. Alloys Compd. 695 **(**2017) 1778-1785.

18. J.A. de Lima Perini, M. Perez-Moya, and R.F.P. Nogueira, Photo-Fenton degradation kinetics of low ciprofloxacin concentration using different iron sources and pH, J. Photochem. Photobio. A 259 **(**2013) 53-58.
